# The Potential of Bioactive Plant Phytochemicals, Pro-Resolving Anti-Inflammatory Lipids, and Statins in the Inhibition of Intervertebral Disc Degeneration, Low Back Pain Resolution, Disc Functional Repair, and Promotion of Intervertebral Disc Regeneration

**DOI:** 10.3390/cells14221758

**Published:** 2025-11-10

**Authors:** James Melrose

**Affiliations:** 1 Raymond Purves Bone and Joint Research Laboratory, Kolling Institute of Medical Research, Northern Sydney Local Health District, University of Sydney at Royal North Shore Hospital, St. Leonards, NSW 2065, Australia; james.melrose@sydney.edu.au; 2 Graduate School of Biomedical Engineering, University of New South Wales, Sydney, NSW 2052, Australia

**Keywords:** flavonoids, terpenoids, glycosides, polyphenolics, alkaloids, inhibition of disc degeneration, disc repair, disc regeneration, statins, proactive lipids

## Abstract

**Highlights:**

**What are the main findings?**

- Flavonoids, terpenoids, glycosides, alkaloids, and polyphenolics show promise in the treatment of intervertebral disc degeneration and low back pain.
- Pro-resolving anti-inflammatory lipids (lipoxin A4, resolvin D1, protectins, and maresins) and statins show promise in the inhibition of intervertebral disc degeneration and promote repair processes.

**What is the implication of the main finding?**

- Many plant compounds show potential in the repair of the intervertebral disc.
- Biological therapies for the treatment of disc disease warrant further investigation.

**Abstract:**

This comprehensive narrative review of bioactive plant compounds, pro-resolving anti-inflammatory lipids, and statins shows their potential in the inhibition of intervertebral disc degeneration (IVDD), pain resolution, tissue repair, and disc regeneration. IVDD is a multifactorial disease involving a multitude of signaling pathways, leading to the loss of normal disc function. An influx of nociceptive mechanoreceptors generate low back pain (LBP). IL6 and IL8 levels are elevated in patients undergoing spinal fusion to alleviate LBP, indicating these pro-inflammatory mediators may be major contributors to the generation of LBP. Apoptosis of disc cells leads to the depletion of key extracellular matrix components that equip the disc with its weight-bearing properties. A biomechanically incompetent degenerated IVD stimulates nociceptor mechanoreceptor activity, generating pain. Myo-tendinous, vertebral body, muscle, and facet joint tissues also contain pain receptors. Disturbance of the normal architecture of the IVD also generates pain in these tissues. Plant compounds have been used in folkloric medicine for centuries. This review attempts to provide a scientific basis for their purported health benefits; however, further studies are still required to substantiate this. Until this evidence is available, it would be prudent to be cautious in the use of such compounds. A diverse range of plant compounds (flavonoids, terpenoids, glycosides, alkaloids, and polyphenolics) inhibit inflammation and apoptosis, reduce spinal pain, and stimulate tissue repair by targeting cell signaling pathways in IVDD. Pro-resolving lipid mediators (lipoxin A4, resolvin D1, protectins, and maresins) also reduce inflammation, maintaining disc health and function. Cholesterol lowering statins disrupt phosphorylation in cell signaling pathways inhibiting IVDD, promoting tissue repair and regeneration.

## 1. Introduction

The intervertebral disc (IVD) is a major contributor to the weight-bearing properties and flexibility of the spine [1], and when it degenerates, it is a major contributor to the generation of low back pain (LBP) due to the mechanical sensitization of pain-generating nerves and mechanoreceptors that grow into the degenerated IVD [2]. LBP is the most impactful of any musculoskeletal condition. A 10-year global study of 291 major human diseases acknowledged that LBP was the most consequential musculoskeletal condition [3,4], and ~80% of the general population will be affected by LBP some time in their life, with sufficient severity to warrant intervention by a physician [5]. It is estimated that about 40% of the world’s human population suffers from LBP [6] (632 million). A total of 5.5% of these have symptomatic intervertebral disc degeneration (IVDD), which contributes to this condition [7]. LBP has been the leading cause of years lived with disability since 1990, and its treatment remains a significant global public healthcare challenge and a major strain on healthcare resources.

Identification of specific bioactive molecules in plant materials in traditional folkloric medicine is confounded by the complex mixture of bioactive compounds present in such preparations. Advances in functional analytical procedures for plant compounds using computational molecular docking [8,9,10,11], computer-based AI design for biomolecule assembly, and pharmaceutical network analysis for complex medicinal products [12,13,14,15,16,17,18,19], plant genomics, and informatics [20], has improved the identification of roles of specific compounds in these healing procedures. A number of bioactive plant compounds have potential uses in the treatment of IVDD [21,22,23,24]. Plants have been used for healing purposes for centuries [25,26] in traditional Chinese and Indian medicine over the last 3000 years [27]. Botanical products with antioxidant, anticarcinogenic, antiallergenic, anti-inflammatory, antimutagenic, and antimicrobial activities have been harnessed in a diverse array of biomedical applications stemming from original observations gleaned in folkloric medicine [28,29,30,31].

Specific aims of this study.

The aim of this narrative review is to illustrate the potential of bioactive plant compounds, pro-resolving anti-inflammatory lipids, and statins as potential therapeutic agents for the treatment of IVDD and LBP. This is the number one musculoskeletal condition and has global socioeconomic significance.

### 1.1. The Complexity of IVDD and Natural Plant-Based Therapeutic Interventions

With IVDD, a number of proinflammatory mediators and cytokines, such as interleukin-6 (IL-6), interleukin-8 (IL-8), prostaglandin E2 (PGE2), nitric oxide (NO), and matrix metalloproteases (MMPs), are produced, leading to tissue inflammation, tissue destruction, and stimulation of nociceptors, resulting in the generation of pain [32,33,34]. Tissue destruction due to elevated MMP levels leads to the depletion of key ECM components, which provides the IVD with its weight-bearing properties. Degenerate disc tissue thus has decreased biomechanical competence, resulting in increased load being transmitted to nociceptors and mechanoreceptors, exacerbating pain generation. IL6 and IL8 levels are elevated in patients undergoing spinal fusion to alleviate LBP, indicating that these pro-inflammatory mediators produced in the NP may be major contributors to the generation of LBP [35]. The effects of inflammatory mediators may be more dominant in the aging spine [36], which is consistent with elevated levels of LBP with ageing. Adults aged 50 years and older are especially vulnerable to LBP [37], with its prevalence increasing up to 80 years of age. Studies show that up to 85 percent of people will experience some form of LBP some time in their lifetime [38].

Degeneration of the IVD is a multifactorial disease of considerable complexity. At least ten cell signaling pathways have roles in IVD degenerative processes [39,40,41,42,43,44,45] (Table 1). A diverse range of plant compounds can target specific aspects of these cell signaling pathways. Many of these plant compounds are ingested as dietary components or as nutritional supplements and are processed by gut bacteria into smaller, more bioavailable metabolites [46]. A gut–IVD axis has been demonstrated [47,48,49], and presumably, blood vessels serving the gut epithelium and lumbar arteries arising from the aorta transport these to the IVD, where they gain access to the IVD by diffusive processes [50]. Degenerated IVDs are depleted of aggrecan, and their collagen networks are damaged [51], resulting in the diminishment of IVD properties, which normally exclude diffusive entry of metabolites, making degenerated IVDs more accessible to small metabolites.

### 1.2. General Comments on Plant Therapeutic Healthcare Products

Five classes of plant compounds have found applications in biomedicine: flavonoids, terpenoids, polyphenolics, glycosides, and alkaloids (Table 2).

#### 1.2.1. Flavonoids

**Figure 1:**
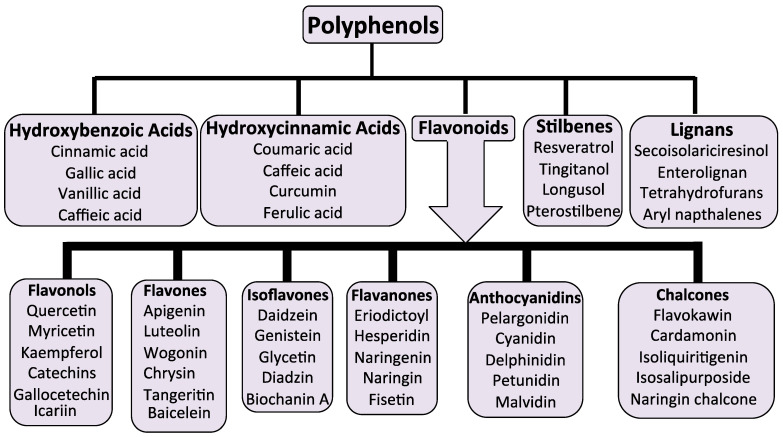
The biodiversity of phenolic plant compounds and their sub-classes.

Over 10,000 flavone/flavonoid compounds have been characterized so far [97]. Plant phenolic compounds are also a biodiverse family of plant compounds (Figure 1). The majority of these have therapeutic properties as anticancer, antimicrobial, antiviral, antiangiogenic, antimalarial, antioxidant, neuroprotective, antitumor, and anti-proliferative agents [98,99,100,101]. Many flavonoid and phenolic compounds have found application in the treatment of IVDD by inhibiting degenerative processes and promoting tissue repair and regeneration [24,102].

#### 1.2.2. Terpenoids

Terpenoids, phenolics, alkaloids, and glycosides display a diverse range of properties in cell signaling pathways that affect inflammation, apoptosis, ECM stabilization, cell viability, and autophagy [103,104,105,106,107,108,109,110]. Terpenoids display an immense level of structural diversity, with at least 40,000 structural presentations identified. Some terpenoids have potent anti-inflammatory properties [104,111] and are volatile components of essential oils, imparting distinctive aromatic signatures to plants such as eucalyptus, peppermint, lemon, pine, lavender, and various herbs. The volatile nature of these compounds provides a novel drug delivery system in aromatherapy to treat inflammatory diseases [103,112]. The few terpenoids that have been used to treat IVDD are discussed in detail later in this review [24,113].

#### 1.2.3. Glycosides

Phenolic acid and flavonoid glycosides form a varied class of naturally occurring compounds with potent anti-oxidant and anti-inflammatory properties and are more soluble than their aglycone forms and more bioavailable, making them more effective interactive molecules [109]. Glycoside flavonoids are transported in the gut by active transport, whereas their aglycone forms are transported through the gut by passive diffusion. Glycosides are also more soluble [114]. Glycosylation can also reduce any toxic effects that may be evident in some aglycone forms of these molecules, and in some cases, can improve their bioactivity [108,115,116]. Glycosides exert anti-inflammatory, antioxidative, and anti-apoptotic effects through PI3K/Akt or JAK/STAT signaling. Some glycosides (ginsenosides, notoginsenoside, astragaloside IV, dioscin, kinsenoside, and crocin) are of interest for the treatment of IVDD [58,94,117,118,119,120].

#### 1.2.4. Alkaloids

Naturally occurring alkaloid compounds have been used in global traditional healing practices since ancient times, and many are still in use today [106,107]. Many alkaloids were originally isolated from weedy plants, which have an enhanced ability to colonize disturbed habitats through highly bioactive compounds, giving them greater adaptability to altered growth conditions. Alkaloids can also have toxic properties and require careful initial assessment, but this inherent toxicity has also proved useful in the eradication of infective organisms [121,122]. Present-day use of alkaloid medicines has benefited from the experience gleaned by traditional medical practitioners who first documented the usefulness of alkaloids as medicines [123]. *Nigella sativa* is a good example of a medicinal plant that has been extensively studied and shown to produce a wide range of compounds with pharmacological properties useful in antidiabetic, anticancer, immunomodulator, analgesic, antimicrobial, anti-inflammatory, spasmolytic, bronchodilator, and hepato-, reno-, and gastro-protective applications [124]. *N. sativa* is a herb native to the Mediterranean, North Africa, Middle East, and Western Asia and has been used as a spice and herbal medicine for many centuries. *Moringa oleifera* is another medicinal plant that contains alkaloid compounds of medicinal importance, which display antimicrobial, antitumor, and anti-hypertensive properties. Thiocarbamate alkaloids isolated from *M. oleifera* have been evaluated in clinical trials for type II diabetes, osteoporosis, dyslipidemias, and HIV infection and are cardio-protective [110,125,126]. Some present-day alkaloid medicinal compounds had their origins in medicinal plants. These include well-known compounds such as vincristine, vinblastine, taxol, morphine, ephedrine, colchicine, codeine, cocaine, berberine, and atropine [106]. Newer plant compounds have also been identified with useful pain relief properties. Chuanxiong rhizome from *Conioselinum anthriscoides* has been used to treat low back pain [127] and stroke [128] in traditional Chinese medicine for centuries. Ligustrazine, an alkyl pyrazine, alkaloid isolated from Chuanxiong, relieves pain, suppresses inflammation [129,130,131,132], is chondroprotective [130,133], and protects the IVD from degenerative effects [127].

## 2. Plant Compounds as Therapeutic Agents for the Treatment of IVDD

A large number of plant compounds are antioxidant and anti-inflammatory molecules that provide tissue protection. These have been specifically investigated to assess if they have protective properties over IVD NP cells and the chondrocytes of the CEP in the chemical and weight-bearing environments they are exposed to during IVDD. These are summarized in Table 3, Table 4, Table 5, Table 6 and Table 7.

### 2.1. Flavonoids That Inhibit IVDD and Promote Tissue Repair and Regeneration

**Figure 2:**
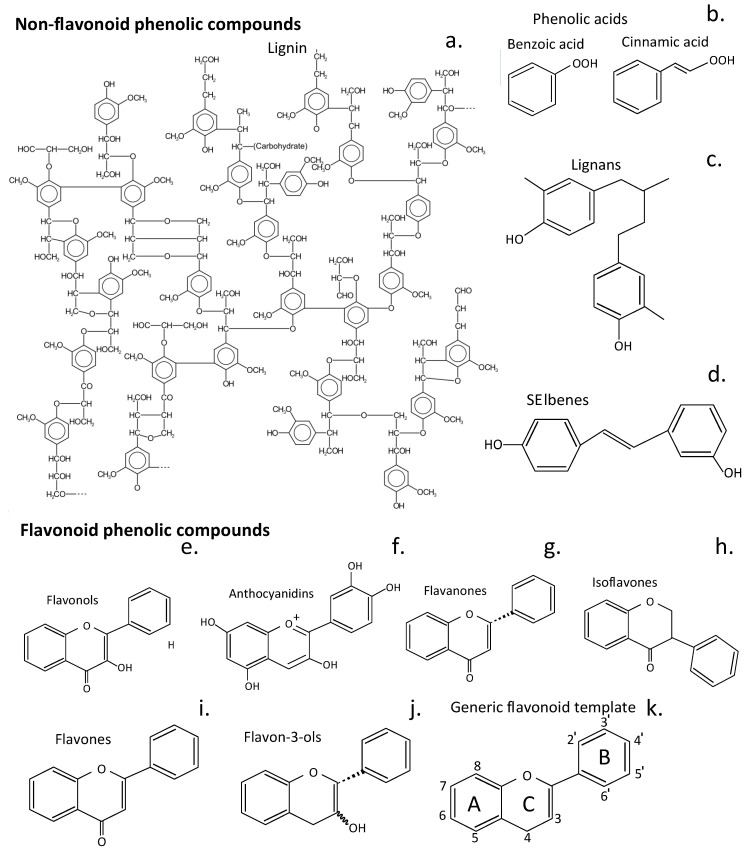
Structures of phenolic plant compound classes that have been used in therapeutic procedures to treat human diseases. An idealized presentation of a soft wood lignin (**a**), phenolic acids (**b**), lignans (**c**), and stilbenes (**d**). Structures of flavonoid phenolic compounds of the sub-classes of flavonoids, flavonols (**e**), anthocyanidins (**f**), flavonones (**g**), isoflavones (**h**), flavones (**i**), and flavan-3-ols (**j**). The generic structure of flavonoids and the numbering system 3-8 and 2^′^-6^′^ for their 3 rings (A, C, B) is shown (**k**). The structures shown are the aglycone structures for each class of compound; however, these compounds also occur as glycosylated isoforms of diverse functions. Glycosylation of flavonoids strongly enhances their water solubility and thus increases their bioavailability [134].

The knowledge base acquired over generations of use of plants in traditional medicine has been invaluable for the identification of plant compounds appropriate for further investigation as prospective therapeutic agents for the treatment of IVDD. Flavonoids are a particularly diverse family of plant compounds with valuable tissue protective and tissue reparative properties, making them appropriate candidates. Flavonoids have been categorized into six sub-classes, as shown in Figure 2e–j, while they are also categorized as polyphenolic compounds and can occur as glycosylated and aglycone forms.

**Table 3:** Flavonoids that have useful properties in the inhibition of IVDD and stimulation of repair and regeneration.

Compound	Mode of Action
Hyperoside	Hyperoside significantly mitigates TNF-α-induced apoptosis in human NP cells by upregulating SIRT1 and Nrf2, and reduces ECM degradation and apoptosis mediated by ER stress [81]. Cell culture data.
Quercetin	Protects NP cells from apoptosis by inhibiting p38 MAPK-mediated autophagy, prevents ECM degeneration and IVDD in a rat tail puncture model [60]. Suppresses apoptosis and ECM degradation through activation of the SIRT1-autophagy signaling pathway [135]. Quercetin is a senolytic agent that binds Keap1-Nrf2 complex and inhibits the NF-κB pathway, reducing the expression of senescence-associated secretory phenotypic factors in IL-1β-stimulated NP cells [136]. Inhibits oxidative stress-induced senescence through the regulation of the miR-34a/SIRT1 axis [137]. Cell culture and animal model data.
Apigenin	Enhances autophagy through the AMP-activated protein kinase (AMPK)/mTOR/transcription factor signaling cascade, alleviates oxidative stress-induced senescence in NP cells, suppresses the expression of TNF-α-mediated pro-inflammatory cytokines, mitigating disc degeneration in rat IVDD models [138]. Animal model data.
Butein	Butein is a chalcone-type flavonoid [139], with antioxidant, anti-inflammatory, antiangiogenic, anticancer, and antidiabetic activities [140]. In vitro and in vivo studies show butein activates SIRT1 and suppresses p53 acetylation, protecting NP cells from apoptosis and senescence triggered by hyperglycemia. Significantly alleviates degenerative effects in diabetic IVDD rat models, where increased NP expression of SIRT1 and decreased p53 acetylation is evident [86]. Cell culture and animal model data.
Baicalein	Baicalein inhibits activation of NF-κB and MAPK signaling, reducing inflammatory cytokine expression, prostaglandin E2 (PGE2), TNF-α, and IL-6 in IL-1β-stimulated NP cells [141]. Prevents ECM degradation and loss of aggrecan and type II collagen [75]. Baicalin alleviates IVDD by inhibiting the p38 MAPK signaling pathway [62]. Cell culture data.
Kaempferol	Network pharmacology data suggest kaempferol may be a key component of traditional Chinese medicines used to treat IVDD [142]. An injectable kaempferol-loaded fibrin gel used in a rat IVDD model reduced inflammation, promoted aggrecan and type II collagen synthesis, and reduced IVDD [143]. Kaempferol inhibits phosphorylation of ERK1/2, downregulates MMP3 and ADAMTS4 expression, significantly restores cell viability, and reduces ROS accumulation and apoptosis in NP cells [142]. Slows IVDD by modifying LPS-induced osteogenesis/adipogenesis imbalance and inflammatory [62] response [144], induces chondrogenesis in ATDC5 cells through activation of ERK/BMP-2 signaling [145]. Injectable kaempferol-loaded fibrin glue inhibits inflammation in IVDD [143]. Cell culture and animal model data.
Luteolin	Luteolin suppresses MMP13, p53, and p21 expression but promotes CDK2, CDK4, and Col2α1 expression in endplate chondrocytes and alleviates cellular senescence [146]. Luteolin also reduces apoptosis of NP cells and reverses TNF-α-induced senescence and inflammation through activation of SIRT6 and inhibition of NF-κB cell signaling [147]. This prevents progressive degenerative changes in IVD tissues. Cell culture data.
Luteoloside	Luteoloside is the 7 O-glycoside of luteolin. Luteoloside inhibits IL-1β-induced apoptosis and catabolism in NP cells and ameliorates IVDD [148]. Cell culture data.
Naringin and Naringenin	Naringin and its aglycone naringenin are effective anti-inflammatory agents in the treatment of low back pain arising from IVDD [149]. Naringin upregulates expressions of Sox-6, BMP2, and aggrecan in IVD cells isolated from degenerated IVDs but downregulates TNF-α and MMP3 expression and promotes NP cell proliferation recovery from IVDD [150]. Naringin suppresses the NF-κB pathway and p53 expression [151], protects endplate chondrocytes from apoptosis by promoting SIRT3-mediated mitophagy, and suppresses NLRP3 inflammasome activation [87] and its contributions to IVDD [152]. Naringin protects human NP cells against TNF-*α*-induced inflammation, oxidative stress by enhancing autophagic flux via AMPK/SIRT1 activation [70]. Naringin inhibits apoptosis induced by cyclic stretch in AF cells diminishing IVDD by inhibition of ROS/NF-*κ*B signaling [153]. Age-related degeneration of NP cells is lowered by inhibition of IGFBP3 activity [154]. Naringin diminishes autophagy-driven oxidative stress-induced apoptosis in NP cells [155]. Cell culture data.
Icariin	Icariin is a traditional Chinese medicine flavonoid glycoside with diverse pharmacological properties [156] affecting bone, inflammation, cancer, immunity, the cardiovascular system, and CNS [157,158,159,160]. Icariin has NP and CEP cell-protective effects in IVDD through its anti-inflammatory and antioxidant properties and promotes ECM synthesis. Icariin prevents IL-1β-induced apoptosis of NP cells via the PI3K/AKT pathway [55] and H_2_O_2_-induced apoptosis of NP cells via PI3K/Akt signaling [56]. It inhibits IL-1β-induced MAPK and NF-κB cell signaling pathways, reduces secretion of proinflammatory factors and degradative enzymes, and alleviates oxidative stress [161]. Icariin activates the Nrf2/HO-1 pathway to promote mitophagy, inhibit ferroptosis, maintain mitochondrial function, and enhance cell survival [162,163]. Chemokines, such as IGF-1, TGF-β, and SDF-1 are upregulated by icariin-promoting tissue repair [163]. Cell culture data.
Fisetin	Fisetin has antioxidant, anti-inflammatory, anticancer, anti-aging, and nephroprotective properties [65,89,164,165,166]. Fisetin protects NP cells by inhibiting oxidative stress and apoptosis, and maintains ECM structural organization [167] acting through the Nrf2/HO-1 pathway to inhibit oxidative stress-induced ferroptosis, reducing disc cell death [39]. Cell culture data.
Acetacetin	Acacetin is a monomethoxy flavonoid with broad therapeutic potential stemming from its anti-inflammatory, antimicrobial, antioxidant, anticancer, anti-obesity, and cardiovascular protective properties [168,169,170,171,172,173]. Acacetin also mitigates the degeneration of NP cells in vitro and IVD tissues in rat IVDD models. In vitro, acacetin activates the Nrf2 pathway and upregulates antioxidant proteins such as HO-1, NADQO-1, and SOD, inhibiting ROS production, reducing COX-2 and iNOS-mediated inflammation. Acetacetin also inhibits the degradation of aggrecan and type II collagen in IVDD models [174,175]. Inhibition of the phosphorylation of p38, JNK, and ERK1/2 by acacetin moderates degenerative effects on NP cells and significantly ameliorates IVDD in rat puncture IVD models [174]. Animal model and cell culture data.
Orientin	Orientin is an 8-C flavone glucoside of luteolin with antioxidant and anti-inflammatory properties. Orientin downregulates the NF-κB pathway, prevents NF-κB translocation to the nucleus, limiting synthesis of TNF-α, IL-6, and IL-1β by inhibiting IκB kinase [176]. Orientin reduces the expression of iNOS and COX-2, reducing the production of pro-inflammatory mediators, such as NO and prostaglandins. MAPK is targeted by orientin, attenuating the activation of p38 MAPK and JNK, which are crucial for inflammation. Orientin downregulates oxidative ER stress and mitochondrial dysfunction through the AMPK/SIRT1 pathway in rat NP cells in vitro and attenuates IVDD in vivo [69]. Animal model and cell culture data.
Cardamonin	Cardamonin protects NP cells from IL-1β-induced inflammation and catabolism via the Nrf2/NF-κB axis [82]. Cell culture data.
Morin	Morin attenuates pyroptosis of NP cells and ameliorates IVDD via inhibition of the TXNIP/NLRP3/caspase-1/IL-1β signaling pathway [177]. Cell culture data.
Glycitin	Protects against IVDD through antagonizing inflammation and oxidative stress in NP cells [178]. Cell culture data.
Genkwanin	Genkwanin is an O-methylated flavone that regulates IVDD through the ITGA2/PI3K/AKT pathway and by inhibiting apoptosis and senescence [76]. Animal model and cell culture data.
Wogonin	Mitigates IVDD through the Nrf2/ARE and MAPK cell signaling pathways [43]. Animal model and cell culture data.
Isoliquiritigenin	Inhibits IVDD induced by oxidative stress and mitochondrial dysfunction through a PPARγ-dependent pathway [179]. Animal model and cell culture data.
Myrcetin	Myrcetin protects against IVDD through regulation of Nrf2/HO-1/NF-κB signaling. Dihydromyricetin inhibits IVDD through inhibition of NLRP3 inflammasome activation via the Keap1/Nrf2/HO-1 pathway [42] and restores autophagy attenuating IVDD by negative regulation of the JAK2/STAT3 pathway [180]. Animal model and cell culture data.
Hesperidin	Mitigation of oxidative stress-induced ferroptosis in NP cells via the Nrf2/NF-κB axis reduces IVDD [80]. Animal model and cell culture data.
Cyanidin	Procyanidin B3 alleviates IVDD via interaction with the TLR4/MD-2 complex [90]. Proanthocyanidins inhibit the apoptosis and aging of NP cells via the PI3K/Akt pathway, delaying IVDD [52]. Procyanidin C1 ameliorates acidic stress-induced NP degeneration through SIRT3/FOXO3-mediated mitochondrial dynamics [181], attenuates apoptosis of NP cells and IVDD via the JAK2/STAT3 signal pathway [67], and attenuates the high hydrostatic pressure-induced degradation of NP ECM by blocking the Wnt/β-catenin signaling [84]. Cell culture data.
Epigallocatechin 3-gallate and Urolithin A	Suppresses IL-1-induced inflammatory responses in IVD and reduces radiculopathic pain [182], protects H_2_O_2_-induced NP cell apoptosis and inflammation by inhibiting cGAS/Sting/NLRP3 activation [183] and oxidative stress [184]. Urolithin A is a flavonoid metabolite generated from dietary epigallocatechin 3-gallate by gut bacteria. Urolithin A has potent anti-inflammatory and antioxidant properties [185], inhibits TNF alpha induced inflammation [186] and TNF alpha catabolic effects on NP cells and IVDD [187]. Animal model and cell culture data.
Sesamin	Sesamin inhibits LPS-induced inflammation and ECM catabolism in the rat IVD [188]. Intradiscal injection of sesamin protects IVDs from lesion-induced IVDD [189]. Animal model and cell culture data.
Casticin	Casticin is a methoxylated flavonol with some hydroxyl groups in the flavonoid structure replaced by methyl groups. Castacin inhibits LPS-stimulated oxidative stress, inflammation, and ECM degradation by activating the Nrf2/HO-1 signaling axis and indirectly blocks the NF-κB pathway, preventing the progression of IVDD rat models. Casticin promotes the nuclear translocation of Nrf2 and blocks the NF-κB pathway, resulting in decreased levels of iNOS, TNF-α, IL-1β, PGE2, MMP-13, ADAMTS-5, and ROS [190]. Animal model and cell culture data.

Abbreviations: SIRT-1; Keap-1, Nrf2, nuclear factor erythroid 2-related factor 2; ECM, extracellular matrix; ER, endoplasmic reticulum; Kelch-like ECH-associated protein 1 (Keap1); AMPK, AMP-activated protein kinase; p38 MAPK, p38 mitogen-activated protein kinase; NF-κB, nuclear factor kappa-light-chain-enhancer of activated B cells; mTOR, mammalian target of rapamycin; TNF-α, tumor necrosis factor alpha; IVDD, intervertebral disc degeneration; IL-1β, interleukin-1 beta; MAPK, mitogen-activated protein kinase; PGE2, prostaglandin E2; IL-6, interleukin-6; Sox-6, SRY-box 6 transcription factor; BMP2, bone morphogenetic protein; SIRT-1, NAD-dependent deacetylase sirtuin-1; MMP3, matrix metalloprotease 3; ADAMTS4, a disintegrin and metalloproteinase with thrombospondin motifs 4; ROS, reactive oxygen species; CDK, cyclin-dependent kinase; SDF-1, stromal-derived factor; HO-1, heme oxygenase-1; NADQO, NAD(P)H:quinone oxidoreductase 1; p38, mitogen-activated protein kinase; JNK, c-Jun N-terminal kinase; ERK1/2, extracellular signal regulated kinase 1/2; COX-2, cyclooxygenase-2, prostaglandin–endoperoxide synthase 2; iNOS, inducible nitric oxide synthase; PPAR, protease-activated receptor; Wnt, wingless-type MMTV integration site family; TLR4/MD-2 complex, Toll-like receptor-4/MD-2 complex; SIRT3/FOXO3, NAD-dependent deacetylase sirtuin-3/Forkhead box O3 protein; LPS, lipopolysaccharide; Nrf2/ARE, Nuclear factor erythroid 2-related factor 2/antioxidant response elements; ITGA2/PI3K/AKT, integrin alpha 2/phosphatidylinositol 3-kinase/protein kinase B; AMPK, 5’AMP-activated protein kinase; iNOS, inducible nitric oxide synthase.

**Figure 3:**
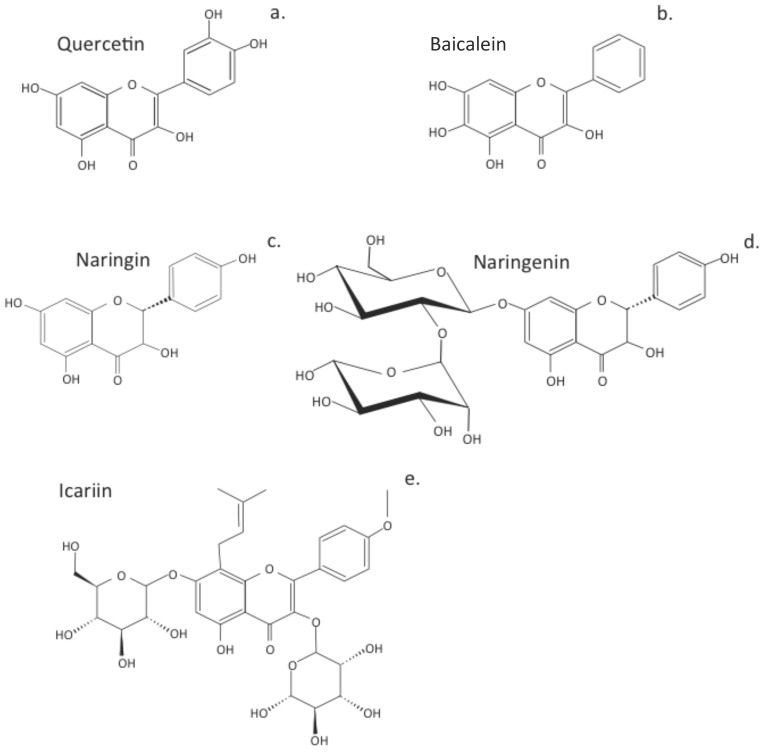
Structures of four flavonoids that show promise for the treatment of intervertebral disc disease. Quercetin (**a**), Baicalein (**b**), Naringin (**c**), Naringenin (**d**) and Icariin (**e**).

Four widely published flavonoids (quercetin, baicalein, icariin, and naringin, Figure 3) were selected for more detailed discussion.

#### 2.1.1. Quercetin as an IVD Therapeutic Agent

Quercetin has been extensively investigated for therapeutic medical purposes [191]. Quercetin is a plant flavonol from the flavonoid group of polyphenols and, like all flavonoids, has potent antioxidant and anti-inflammatory properties, which are the basis of many of its medicinal properties (Figure 3a). Quercetin is a widely distributed pigment in fruits, vegetables, teas, wine, and cereals and one of the most abundant antioxidants in the human diet. Quercetin has important roles in preventing ROS damage to tissues. Quercetin inhibits p38 MAPK signaling, an enzyme of the serine and threonine (Ser/Thr) protein kinase family, associated with the progression of IVDD [64]. Promotion of cell apoptosis and senescence suppresses cell proliferation and autophagy. p38 MAPK thus represents a logical therapeutic target in the prospective treatment of IVDD. However, p38 MAPK is but one cell signaling pathway operative during IVDD. Quercetin protects NP cells from apoptosis by inhibiting p38 MAPK-mediated autophagy, and prevented IVDD in a rat tail IVD puncture model [60]. Quercetin also suppresses apoptosis and ECM degradation through activation of the SIRT1-autophagy cell signaling pathway [135]. With aging, the IVD loses hydration and structural integrity, resulting in elevation in cell tress levels that can lead to a dysfunctional state known as cellular senescence [192,193,194,195]. Senescent cells are metabolically active, but their cycle cell has ceased, and the cells are unable to divide. However, they release a range of damaging molecules in a senescence-associated secretory phenotype. Quercetin is a senolytic agent that binds Keap1-Nrf2 complex and inhibits the NF-κB pathway, reducing the expression of senescence-associated secretory phenotypic factors in IL-1β-stimulated NP cells [136]. Inhibition of oxidative stress-induced senescence by quercetin is also achieved through regulation of the miR-34a/SIRT1 axis [137]. Thus, quercetin is a multi-functional IVD protective compound.

#### 2.1.2. Protective Roles for Baicalein in IVD Tissues

Baicalein has a similar structure to quercetin but is not hydroxylated on the 3′ and 4′ positions of the flavonoid B ring. It is hydroxylated on position 6 on ring A, which is absent in quercetin (Figure 2k). Baicalein is the aglycone form of baicalin and a natural health supplement originally extracted from the roots of the Chinese herbs *Scutellaria baicalensis* and *Scutellaria lateriflora*. Baicalein’s tissue protective properties stems from its ability to scavenge ROS and to interact with signaling molecules operative in apoptosis, inflammation, autophagy, and mitochondrial dynamics. Baicalein reduces inflammatory cytokine expression by inhibiting the activation of NF-κB and p38 MAPK cell signaling [62]. This also reduces prostaglandin E2 (PGE2), TNF-α, and IL-6 production in IL-1β-stimulated NP cells [141] and prevents ECM degradation and loss of aggrecan and type II collagen from the IVD [75].

#### 2.1.3. Naringin and Naringenin Therapeutic Properties in the Treatment of IVDD

Citrus fruits are rich sources of naringin and naringenin. These are flavonoids with strong anti-inflammatory and antioxidant activities. Naringin and naringenin prevent the oxidation of LDL and reduce total cholesterol and HDL levels, but LDL, VLDL, and tri-glycerol levels are unaffected, showing their potential in the treatment of hyperlipidemia. Naringin and its aglycone naringenin are also effective anti-inflammatory agents in the treatment of LBP arising from IVDD [149]. Naringin upregulates the expression of Sox-6, BMP2, and aggrecan in IVD cells isolated from degenerated IVDs but downregulates TNF-α and MMP3 expression and promotes NP cell proliferation and recovery from IVDD [150]. Naringin suppresses the NF-κB pathway and p53 expression [151], demonstrating its anti-inflammatory properties. This protects endplate chondrocytes from apoptosis by promoting SIRT3-mediated mitophagy and suppresses NLRP3 inflammasome activation [87] and IVDD [152]. Naringin protects human NP cells against TNF-*α*-induced inflammation and oxidative stress by enhancing autophagic flux via AMPK/SIRT1 activation [70] and inhibits apoptosis induced by cyclic stretch in AF cells, diminishing IVDD and ROS/NF-*κ*B signaling [153]. Naringin diminishes autophagy-driven oxidative stress-induced apoptosis in NP cells [155].

#### 2.1.4. Icariin and Its Potential Roles in the Treatment of IVDD

Horny goat weed and some herbs are sources of icariin [196]. Icariin is a prenylated flavonol traditional Chinese medicine with diverse pharmacological properties [156] affecting bone, inflammation, cancer, immunity, the cardiovascular system, and CNS [157,158,159,160]. Icariin prevents IL-1β-induced apoptosis of NP cells via the PI3K/AKT pathway [55] and H_2_O_2_-induced apoptosis of NP cells via PI3K/Akt signaling [56]. Inhibition of IL-1β-induced MAPK and NF-κB cell signaling pathways by icariin reduces the secretion of proinflammatory mediators, degradative enzymes, and alleviates oxidative stress [161]. Icariin activates the Nrf2/HO-1 cell signaling pathway to promote mitophagy, inhibit ferroptosis, maintain mitochondrial function, and enhance cell survival [162,163]. Chemokines such as IGF-1, TGF-β, and SDF-1 are upregulated by icariin, promoting tissue repair [163]. Ferroptosis is a type of programmed cell death that is dependent on iron and characterized by the accumulation of lipid peroxides [197]. It is distinct from other forms of regulated cell death, such as apoptosis and necroptosis.

## 3. Terpenoids Displaying Potential in Tissue Protection and Treatment of IVDD

**Figure 4:**
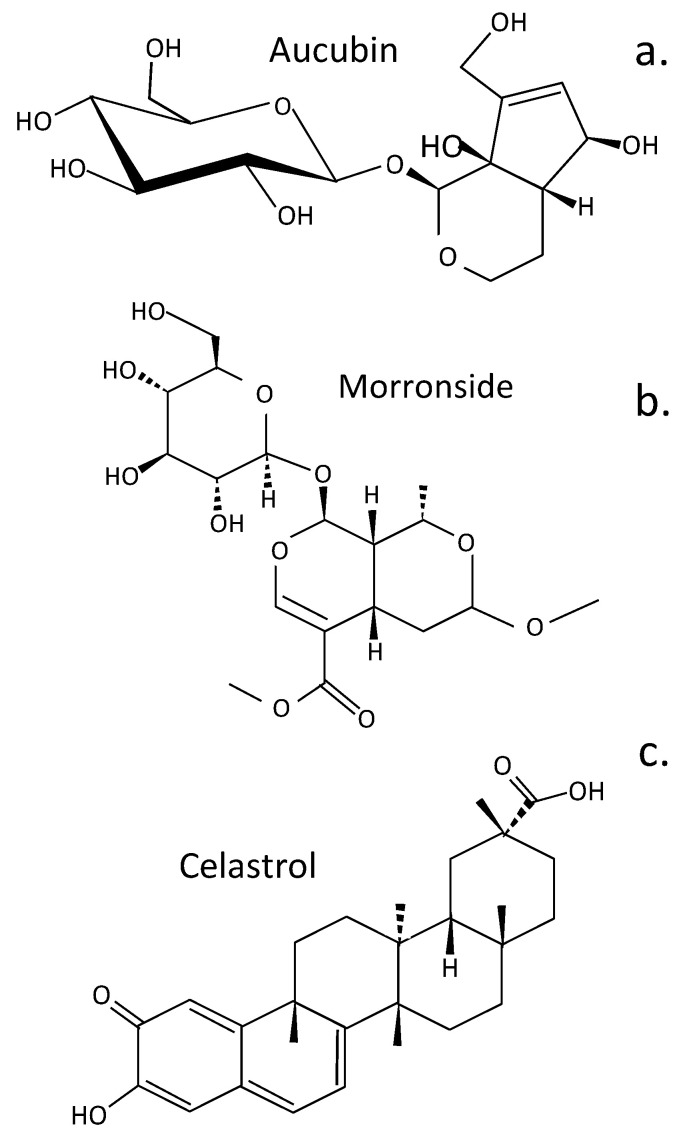
Structure of terpenoids that have been evaluated for the treatment of IVDD. Aucubin (**a**), Morronside (**b**), Celastrol (**c**).

Terpenoids are diverse natural secondary plant metabolites formed from isoprene units (C-5) (Figure 4). These have a wide range of biological properties, including antioxidant, antimicrobial, anti-inflammatory, antiallergic, anticancer, antimetastatic, antiangiogenic, and apoptotic properties [198]. Some terpenoids (aucubin, morronside, and celastrol) have biological properties applicable to the treatment of IVDD. Aucubin represses the activation of the NF-κB-NLRP3 inflammasome in chondrocytes in the CEP [199]. Aucubin is an iridoid glycoside that has extensive biological properties as an antioxidant, anti-aging, anti-inflammatory, anti-fibrotic, anticancer, hepatoprotective, neuroprotective, and osteo protective agent [200]. Morronside is a traditional Chinese herbal preparation that has been used for centuries in traditional medical practice to protect the nervous system, treat OA, inhibit platelet aggregation, prevent diabetic angiopathies and renal damage, and reduce bone resorption [201]. NP cell senescence is also attenuated, alleviating IVDD via inhibition of the ROS-Hippo-p53 cell signaling pathway [202]. Celastrol is a pentacyclic nortriterpene quinone member of the terpenoid family, which has anti-inflammatory, antioxidant, and anticancer activities. Celastrol has been applied to the treatment of chronic inflammatory and autoimmune diseases (e.g., RA, MS, SLE, inflammatory bowel disease, and psoriasis) [203]. Kongensin A is a diterpene plant product isolated from *Croton kongensis*, a tropical shrub, which is a potent inhibitor of necroptosis and an inducer of apoptosis [204]. Kongensin A covalently binds to HSP90, dissociating it from its co-chaperone CDC37, leading to the inhibition of receptor-interacting serine/threonine-protein kinase 3 (RIP3)-mediated necroptosis and the promotion of apoptosis in multiple cancer cell lines [205]. Kongensin inhibits mitogen-activated protein kinase kinase kinase 7 (TAK1, Map3k7), a key regulator of innate immunity, cell death, inflammation, and cellular homeostasis. Kongensin upregulates TAK1 expression in NP cells during IVDD, inhibiting PANoptosis by suppressing oxidative stress. This delays IVDD progression and is a promising novel therapeutic approach to the treatment of IVDD [91].

**Table 4:** Terpenoids with useful therapeutic properties for the treatment of IVDD.

Compound	Therapeutic Properties in IVDD
Aucubin	Represses NF-κB-NLRP3 inflammasome activation in CEP chondrocytes [199]. Cell culture data.
Morroniside	Attenuates NP cell senescence to alleviate IVDD via inhibition of the ROS-Hippo-p53 pathway [202]. Cell culture data.
Celastrol	Reduces IL-1β-induced ECM catabolism, oxidative stress, and inflammation in NP cells attenuating rat IVDD in vivo [206]. Animal model and cell culture data.
Kongensin	Kongensin upregulates TAK1 expression in NP cells during IVDD and inhibits PANoptosis, suppressing oxidative stress delaying IVDD progression [91]. Animal model and cell culture data.

## 4. Phenolic Compounds Displaying Potential as IVD Protective Agents

**Figure 5:**
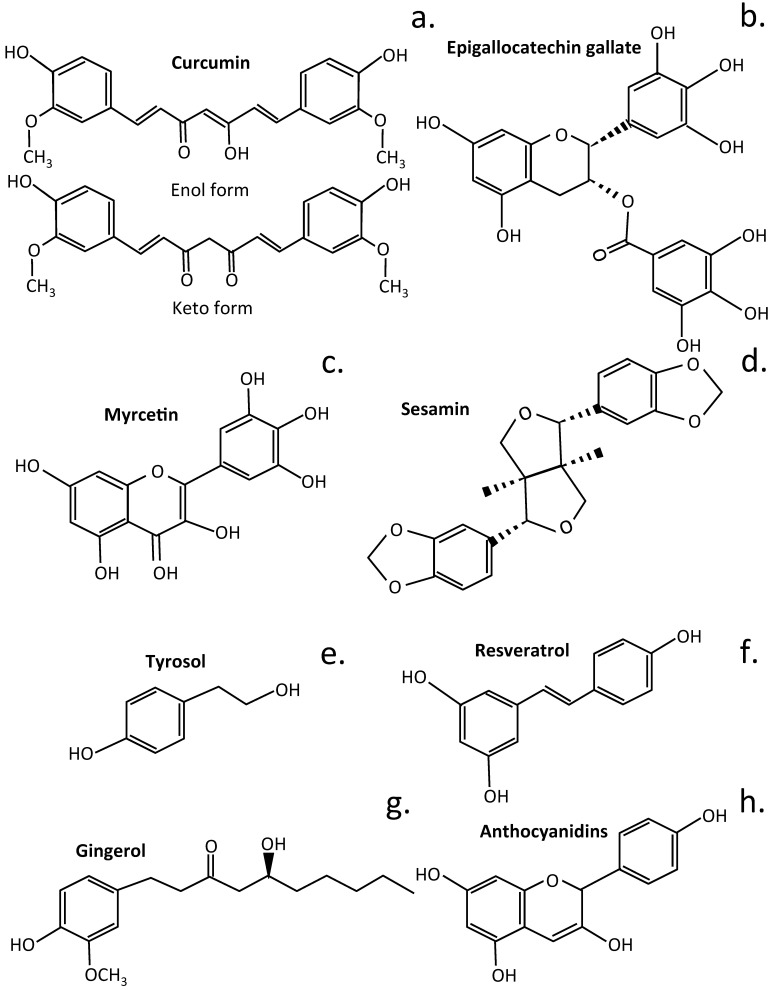
Structures of some phenolic plant compounds that have been used to treat IVDD. Curcumin in its enol and keto forms (**a**), Epigallocatechin gallate (**b**), Myrcetin (**c**), Sesamin (**d**), Tyrosol (**e**), Resveratrol (**f**), Gingerol (**g**), Anthocyanidins (**h**).

Polyphenolic (derived from the Greek word polus, meaning many) compounds are widely distributed in the plant kingdom and are a large family of structurally diverse molecules spanning phenolic acids, flavonoids, tannic acid, stilbenes, lignans, lignins, and ellagitannins [207]. Polyphenolics have broad antioxidant and anti-inflammatory properties (Figure 5, Table 5).

Curcumin attenuates NF-κB expression [208] and regulates iNOS, COX-2, TGF-β1/2, MMP-9, and BDNF in IVDD [41], thus producing anti-inflammatory and anti-catabolic effects on human IVD cells [209]. Curcumin also has a protective effect on endplate chondrocytes, inhibiting IL-1β-induced apoptosis mediated through Bcl-2/Bax [210]. Myricetin is a member of the flavonol class of flavonoids but is also classified as a polyphenolic compound. Myrcetin attenuates IVDD through its regulatory effects over the Nrf2/HO-1/NF-κB cell signaling pathway [211]. Sesamin inhibits inflammation induced by LPS, preventing degradative events that lead to catabolism of key function IVD components [188]. Intradiscal injection of sesamin prevents ECM catabolism in animal models of lesion-induced IVDD by upregulation of the macroautophagy and autophagy family member Beclin 2 (BECN2). This is a regulator of G-protein coupled receptor turnover, which facilitates phosphatidylinositol 3-kinase binding and activity in the phosphatidylinositol 3-kinase-GPRASP1 (G Protein-Coupled Receptor Associated Sorting Protein 1) complex as a regulator of autophagy and as a regulator of G-protein-coupled receptor turnover [189]. Sesamin also ameliorates CEP degeneration in models of IVDD [212].

Dried green tea leaves have a high content of Epigallocatechin Gallate (EGCG), white tea has appreciable levels, and black tea has lower EGCG levels. EGCG has potent regulatory properties over molecular pathways that control inflammation, oxidative stress, and apoptosis [213,214,215]. EGCG has protective effects over IVD cells and provides protection from oxidative stress [184]. It also suppresses IL-1β-induced inflammatory responses, reduces radiculopathic pain [182], and protects the IVD from H_2_O_2_-induced NP cell apoptosis and inflammation by inhibiting cGAS/Sting/NLRP3 activation [183] and the activation of the NLRP3 inflammasome and secretion of IL-1β [216]. The EGCG component of green tea is a potent inhibitor of fatty acid synthase [217]. Low bioavailability of tea catechins, however, can limit their effectiveness as therapeutic agents. Methods have therefore been investigated to depolymerize EGCG into metabolites with improved bioavailability using enzymatic treatment with tannase and pectinases [218]. The gut microbiota also transform EGCG into more bioavailable phenolic metabolites that retain or actually have improved biological activity [46,219,220,221]. Fermentation of green tea to form black tea also results in the bioconversion of EGCG into smaller bioactive metabolites [222]. Urolithins are generated from EGCG by the gut microbiota [46,223], and these have greater bioavailability and anti-inflammatory, antioxidant, antitumor, and anti-aging properties than EGCG [223]. γ-Valerolactones are also generated by gut bacteria by the bioconversion of EGCG [224].

Resveratrol has potent antioxidant and anti-inflammatory properties in the laboratory and similar protective properties in tissues, provided that therapeutic doses are achieved. Resveratrol stimulates anabolic processes in the IVD, stimulating IVDD repair [225,226,227,228]. Anthocyanin is the glycoside form of anthocyanidin and a specific type of berry fruit flavonoid of elderberry, chokeberry, black raspberry, bilberry, blackcurrant, and blueberry [229,230]. Anthocyanidins are responsible for the blue, red, and purple coloration of flowerheads and berry fruits [229]. Anthocyanins undergo phase II reactions in the gut, where the aglycone anthocyanidin is subjected to methylation, sulphation, and glucosidation. The gut microbiota then transform the anthocyanins into protocatechuic acid [231] and phlorglucinaldehyde (1,4,6-trihydroxy benzaldehyde) metabolites, which have greater bioavailability [232]. Cyanidins attenuate NP cell apoptosis in IVDD through the JAK2/STAT3 cell signaling pathway [67], protect against high glucose-induced injury in human NP cells by regulating Nrf2/HO-1 signaling [96], and attenuate high hydrostatic pressure-induced ECM degradation by blocking Wnt/β-catenin signaling [84]. Tyrosol is a biophenol secondary metabolite found in olive oil and wine with antioxidant, stress-protective, and anti-inflammatory properties [233]. It forms part of the health-promoting Mediterranean diet [234]. Tyrosol upregulates Sirt1 expression, suppressing apoptosis and inflammation in IL-1β-stimulated human NP cells through activation of the PI3K/Akt pathway [74]. Gingerol is a phenolic compound with antioxidant, antitumor, and anti-inflammatory properties of general application in the treatment of chronic diseases [235], and it also attenuates IVDD by inhibiting IL-1β-mediated NLRP3 cell signaling [236].

**Table 5:** Phenolic compounds with therapeutic properties for the treatment of IVDD.

Compound	Therapeutic Properties in IVDD
Curcumin	Attenuates NF-κB expression in rat lumbar IVDD [208], regulates the expression of iNOS, COX-2, TGF-β1/2, MMP-9, and BDNF in a rat model of IVDD [41], exhibits anti-inflammatory and anti-catabolic effects on human IVD cells by reducing TLR2 expression and JNK activity [209], and protects rat CEP chondrocytes from IL-1β-induced apoptosis via Bcl-2/Bax regulation [210]. Animal model and cell culture data.
Myrcetin	Myricetin is structurally similar to fisetin, luteolin, and quercetin and is reported to have many of the same functions as these other members of the flavonol class of flavonoids but is also a polyphenolic compound. Myrcetin attenuates IVDD through regulation of the Nrf2/HO-1/NF-κB signaling pathway [211]. Animal model and cell culture data.
Sesamin	Inhibits LPS-induced inflammation and ECM catabolism in rat IVD [192]. Animal model and cell culture data.
Epigallocatechin Gallate	Epigallocatechin gallate protects IVD cells from oxidative stress [184]. Epigallocatechin 3-gallate suppresses interleukin-1β-induced inflammatory responses in IVD cells, reduces radiculopathic pain [182], and protects the IVD from H_2_O_2_-induced NP cell apoptosis and inflammation by inhibiting cGAS/Sting/NLRP3 activation [183]. Animal model and cell culture data.
Resveratrol	Has antioxidant and anti-inflammatory properties inhibiting IVDD and stimulates anabolic properties in IVD repair [225,226,227,228]. Animal model and cell culture data.
Anthocyanidins	Cyanidin attenuates the apoptosis of rat NP cells and IVDD via the JAK2/STAT3 signal pathway [67]. Cyanidin-3-glucoside protects against high glucose-induced injury in human NP cells by regulating Nrf2/HO-1 signaling [96]. Cyanidin attenuates high hydrostatic pressure-induced ECM degradation by blocking Wnt/β-catenin signaling [84]. Animal model and cell culture data.
Tyrosol	Upregulates Sirt1 expression, suppresses apoptosis and inflammation, and modulates ECM remodeling in IL-1β-stimulated human NP cells through activation of the PI3K/Akt pathway [74]. Cell culture data.
Gingerol	Ameliorates IVDD by inhibiting IL-1β-mediated NLRP3 cell signaling [236]. Cell culture data.

## 5. Alkaloids Displaying Therapeutic Potential in the Treatment of IVDD

**Figure 6:**
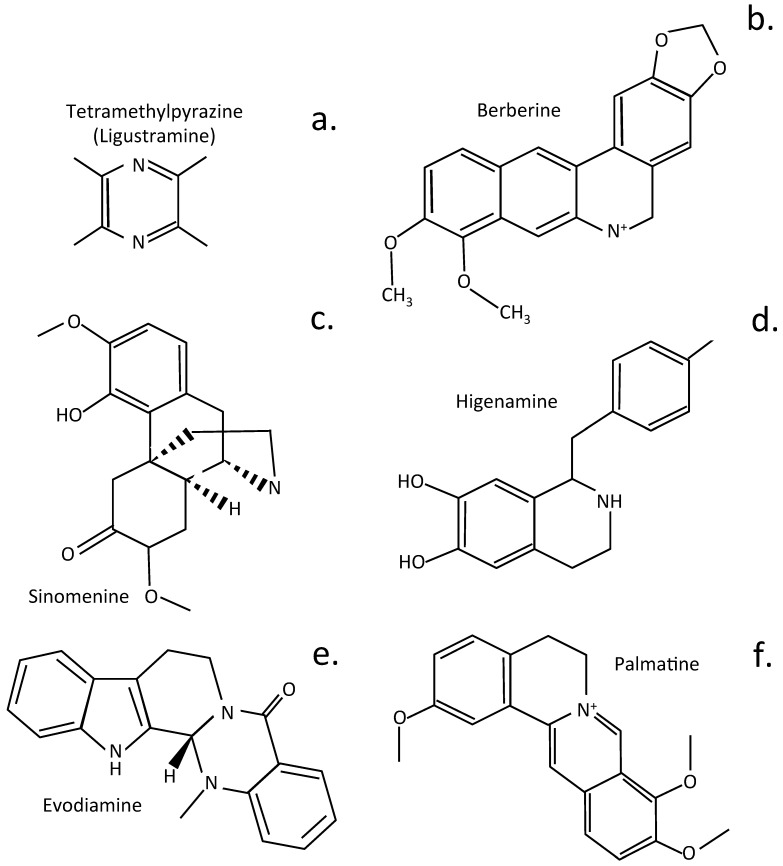
Structure of alkaloid plant compounds that have been used to treat IVDD. Ligustramine (**a**), Berberine (**b**), Sinomenine (**c**), Higenamine (**d**), Evodiamine (**e**), Palmatine (**f**).

Ligustrazine (tetramethylpyrazine) is a small alkyl pyrazine plant compound of the Chinese herb *ligusticum chuanxiong hort* (chuanxiong). It has been used for centuries in traditional Chinese medicine to treat LBP. In more recent times, ligustrazine has been identified as a useful molecular template amenable to chemical modifications to produce customized pharmaceutical compounds for specific human diseases [237,238] (Figure 6). Ligustrazine has been used to provide pain relief in knee OA [130]. Ligustrazine also suppresses aberrant TGF*β* activation in NP cells, preventing the development of IVDD [239]. It also has IVD protective properties [127] and inhibits CEP hypertrophy via suppression of TGF-β1 activity in models of IVDD [240].

Berberine is a bioactive herbal quaternary ammonium salt of the protoberberine group of isoquinoline alkaloids and has anti-inflammatory properties useful for application in a number of chronic disease [241,242,243,244,245]. Berberine has protective properties over disc cell populations in disease, ameliorates oxidative stress and its effects, resulting in induction of apoptosis by modulating ER stress and autophagy in human NP cells [246]. Suppression of apoptosis and ECM degradation in NP cells ameliorates IVDD in the intact IVD [92]. In human NP cells, berberine inhibits the NFκB pathway, preventing the development of inflammatory conditions in the disc. It also has protective properties, shielding intervertebral disc cells from the effects of IL-1β-induced ECM degradation, altered IVD mechanics, and the induction of apoptosis [247].

Sinomenine is an alkaloid isolated from *Caulis sinomenii*, and *Sinomenium acutum* and has been used for 30 years in traditional Chinese medicine as an anti-inflammatory drug [248]. Sinomenine displays bioactivities relevant to the treatment of RA and alleviation of inflammation [249]. Sinomenine also ameliorates IL-1β-induced IVDD in rat models through suppression of inflammation and oxidative stress, mediated by the Keap1/Nrf2/NF-κB cell signaling pathway [83]. It also inhibits apoptosis and autophagy in vitro and in vivo [250].

The root of the Chinese herbal plant called Fuzi, (*Radix Aconiti lateralis praeparata*) is a source of the alkaloid drug higenamine. This is a widely used drug in traditional Chinese medicine to treat various cardiovascular and skeletal medical disorders [251]. Higenamine inhibits IL-1β-induced human NP cell apoptosis by mediating ROS free radical effects through PI3K/Akt signaling [71]. It also inhibits IL-1β-induced inflammation in human NP cells [252]. Higenamine also inhibits acute and chronic inflammatory pain through the modulation of TRPV4 channels; thus, its therapeutic value in the treatment of IVDD may be enhanced by its ability to prevent the generation of LBP [253].

Evodiamine is a quinolone alkaloid isolated from the fruit of *Evodia rutaecarpa*. This is a drug with broad application in folkloric Chinese medicine and is a popular traditional Chinese herb [254]. Evodiamine has also been shown to ameliorate IVDD through inhibitory effects on Nrf2 and MAPK cell signaling pathways [61] and by the activation of the PI3K/AKT cell signaling pathway to block IVDD [40].

Palmatine is a natural isoquinoline alkaloid and has a wide range of pharmacological properties [255]. Palmatine activates TFEB [256] (transcription factor EB), enhances autophagy, and alleviates ER stress in IVDD [257]. TFEB is a pivotal transcription factor, with roles in the regulation of lysosomal biogenesis and autophagy [256,258].

**Table 6:** Alkaloids displaying therapeutic properties for the treatment of IVDD.

Compound	Therapeutic Properties in IVDD
Ligustrazine	Suppresses aberrant TGF*β* activation of NP cells to prevent IVDD [239]. It has IVD protective properties [127] and inhibits CEP hypertrophy via suppression of TGF-β1 activity [240]. Cell culture data.
Berberine	Ameliorates oxidative stress-induced apoptosis by modulating ER stress and autophagy in human NP cells [246], suppresses apoptosis and ECM degradation in NP cells, ameliorates IVDD [92], and prevents human NP cells from IL-1β-induced ECM degradation and apoptosis by inhibiting the NFκB pathway [247]. Cell culture data.
Sinomenine	Ameliorates IL-1β-induced IVDD in rats through suppression of inflammation and oxidative stress via Keap1/Nrf2/NF-κB cell signaling [83] and ameliorates IVDD via inhibition of apoptosis and autophagy in vitro and in vivo [250]. Animal model and cell culture data.
Higenamine	Mitigates IL-1β-induced human NP cell apoptosis by ROS-mediated PI3K/Akt signaling [71], inhibits IL-1β-induced inflammation in human NP cells [252]. Cell culture data.
Evodiamine	Ameliorates IVDD through the Nrf2 and MAPK cell signaling pathways [61], activates PI3K/AKT cell signaling pathway to block IVDD [40]. Animal model and cell culture data.
Palmatine	Activates TFEB, enhances autophagy, and alleviates ER stress in IVDD [257]. Cell culture data.

## 6. Glycoside IVDD Treatment Compounds

### 6.1. Ginsenosides

**Figure 7:**
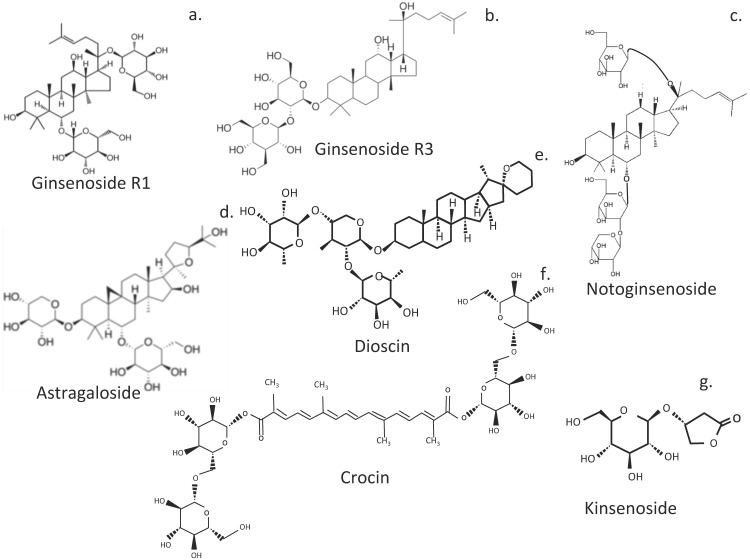
Glycoside plant compounds that show promise in the treatment of IVDD. Ginsenoside R1 (**a**), Ginsenoside R3 (**b**), Notoginsenoside (**c**), Astragaloside (**d**), Dioscin (**e**), Crocin (**f**), Kinsenoside (**g**).

Ginseng is a widely used herbal nutraceutical traditional Chinese medicine [259] and has antioxidant and anti-inflammatory activities [260]. Ginsenoside bioactive components of ginseng are triterpenoid saponins, and more than 180 types have been identified [117] (Figure 7). Ginsenoside Rg1 is of major interest for the treatment of IVDD since it regulates disc homeostasis and its hydration and inhibits apoptosis, inflammation, and ECM degradation, delaying IVDD progression. Rg1 improves the proliferation of NP cells and reduces apoptosis. Prevention of IVDD by Rg1 is affected through inhibition of the Wnt/β-catenin signaling pathway [261] and by suppressing the activation of the Yes-associated protein (YAP)/transcriptional coactivator with the PDZ-binding motif (TAZ-1) transcriptional coactivator Hippo cell signaling pathway. This significantly increases the mechanical strength of IVDs in rat IVDD models [262]. Rg1 prevents the activation of the NF-κB signaling pathway, inhibits apoptosis, suppresses IL-6 and TNF-α expression in IL-1-treated NP cells, and stimulates aggrecan and collagen II biosynthesis, an inhibitor of kappa B kinase (IκK) [263]. Rg3 ginsenoside also reverses IL-1β-induced apoptosis through inactivation of the p38 MAPK pathway. This significantly reduces MMP2, MMP3, ADAMTS-4, and ADAMTS-5 activity in IVDD. However, Rg3 not only addresses degenerative changes in the NP but also restores AF lamellar organization and the functional properties of the IVD as a weight-bearing structure [264].

### 6.2. Notoginsenosides

Notoginsenoside R1 (NR1) suppressed IVDD in a rat annular puncture model, restored IVD functional properties, and suppressed mechanical and thermal hyperalgesia [265]. Moreover, NR1 promoted ECM synthesis in vivo and in vitro and decreased proinflammatory cytokine mRNA expression, inactivated NF-κB/NLRP3 cell signaling pathways, and obviated inflammation in the IVD [266]. A cellular environment in the IVD was thus established by NR1, conducive to NP cellular activity. NR1 protects the IVD from degeneration through suppression of the NF-κB/NLRP3 cell signaling pathway.

### 6.3. Astragaloside IV

Network pharmacology and molecular docking procedures have been used to investigate the mechanism of action of Astragaloside IV in the treatment of IVDD in a lumbar spine IVD instability mouse model [267]. Disc height and volume and matrix metabolism were improved, along with Col2α1 and aggrecan expression. Network pharmacology analysis revealed 11 key core genes, including ALB (albumin gene), MAPK1, MAPK14 (p38 MAPK), EGFR, TGFBR1, MAPK8, MMP3, ANXA5 [268], ESR1, CASP3, and IGF1. ANXA5 has anti-inflammatory properties, is neuroprotective, promotes osteogenic differentiation, and takes part in chondrocyte apoptosis and mineralization [268]. ESR1 encodes an estrogen receptor. IVD protective effects were mediated through inhibition of the EGFR/MAPK signaling pathway.

### 6.4. Dioscin

Dioscin is a multi-targeting bioactive steroid saponin phytocompound from traditional Chinese medicine [269]. Dioscin has shown potential as a prospective therapeutic agent for the treatment of IVDD through attenuation of IL-1β-induced catabolism and apoptosis, mediated by TLR4-NF-κB signaling in human NP cells [94].

### 6.5. Kinsenoside

Kinsenoside, ameliorates IVDD through the activation of the AKT-ERK1/2-Nrf2 cell signaling pathway [58].

### 6.6. Crocin

Crocin, a glycosylated carotenoid of *Crocus sativus* L. (saffron) [120] has antioxidant, anti-inflammatory, neuroprotective, anti-retinopathy, anticancer, and antidepressant properties [85,119,270,271]. Crocin inhibits inflammation and IVDD catabolic processes [118], significantly suppresses LPS-induced overexpression of MMP-1, MMP-3, MMP-13, ADAMTS-4, ADAMTS-5, and proinflammatory IL-1β, TNF-α, IL-6, iNOS, and TLR-2 in-vitro.

**Table 7:** Glycosides displaying therapeutic properties in the treatment of IVDD.

Compound	Therapeutic Properties in IVDD
Ginsenosides	Ginsenoside Rg1 inhibits NP cell apoptosis, inflammation, and ECM degradation via YAP1/TAZ/Hippo cell signaling [262]. Rg1 relieves rat IVDD and inhibits IL-1β-induced NP cell apoptosis and inflammation via NF-κB signaling [261,263]. Ginsenoside Rg3 exhibited anti-catabolic and anti-apoptotic effects in IL-1β-treated human disc NP cells and in a rat model of IVDD by inactivating the MAPK cell signaling pathway [264], ginsenoside Rg3 inhibited NF-κB signaling in TNF-α-stimulated human NP cells inhibiting IVDD [272]. Animal model and cell culture data.
Notoginsenoside	Notoginsenoside R1 suppresses the inflammatory response/pyrop tosis in NP cells via inactivation of NF-κB/NLRP3 cell signaling [265]. Animal model and cell culture data.
Astragaloside IV	Attenuates IL-1β-induced IVDD through inhibition of the NF-κB pathway [273], relieves IL-1β-induced human NP cell degeneration through modulating PI3K/Akt signaling pathway [72], inhibits miR-223/JAK2/STAT1 signaling to alleviate LPS-induced damage in NP [66], activates telomerase activity protecting NP cells from high glucose-induced senescence and apoptosis [274]. Animal model and cell culture data.
Dioscin	Attenuates IL-1β-induced catabolism and apoptosis through modulation of TLR4-NF-κB signaling in human NP cells [94]. Cell culture data.
Kinsenoside	Ameliorates IVDD through the activation of AKT-ERK1/2-Nrf2 signaling pathway [58]. Animal model and Cell culture data.
Crocin	Has anti-inflammatory and anti-catabolic effects on rat IVDs through suppression of JNK signaling activation [118]. Animal model and cell culture data.

## 7. The Role of Pigmented Compounds and Lipids in IVD Bioregulation

### 7.1. Emodin

Emodin is a bioactive anthraquinone that upregulates anabolic markers (COL2A1, aggrecan) and negatively regulates catabolic markers (MMP3, MMP13) in cultured NP cells, inhibiting cell apoptosis in the inflammatory environment of degenerated IVDs [275] and effectively alleviating IVDD in a rat model. Emodin inhibits inflammation-induced NF-ĸB activation through suppression of the degradation of LRP1 via the proteasome pathway [275]. Emodin treatment prevents reduced NP cell viability induced by IL-1β by reducing elevated ROS levels, secretion of IL-6 and TNF-*α*, and caspase-3 activity to abolish IL-1*β*-induced inflammation in NP cells [276]. Emodin is a potent and selective inhibitor of NLRP3 inflammasome activation, suppressing casein kinase II (CK2)-mediated phosphorylation of FUNDC1, a pivotal mitophagy receptor. This prevents mitochondrial ROS-induced NLRP3 inflammasome assembly [88].

### 7.2. Rhein

Rhein (RH, 4,5-dihydroxyanthraquinone 2-carboxylic acid) is a lipophilic anthraquinone that enhances the synthesis of ECM components and inhibits production of inflammatory mediators in the IVD. The metabolic precursor of rhein, diacerein has significant pain-relieving properties in OA and provides functional improvement in joint function. RH may be a useful compound to evaluate for the treatment of IVDD since it has the ability to diminish IL-1-induced apoptosis and secretion of MMPs and aggrecanases [277,278,279,280].

### 7.3. Physcion (Parietin)

Physcion is an anthraquinone from rhubarb that displays antimicrobial, antitumor, antioxidant, and anti-inflammatory properties that counter inflammation and tissue damage [281,282,283].

## 8. Statins, Cholesterol, and Animal Models for Experimental IVDD

Statins are cholesterol-lowering drugs originally isolated from fungi [284,285]. *Monascus* spp., *Penicillium* spp., *Aspergillus terreus*, and *Pleurotus ostreatus* were used to characterize the first naturally occurring statin, mevastatin (compactin) [286,287]. Statin drugs are now produced by pharmaceutical companies and are probably the most frequently prescribed global drug. Besides their cholesterol-lowering properties, statins have beneficial effects in the treatment of IVDD, inhibiting degenerative changes in the IVD and stimulating repair [288,289,290]. Intradiscal administration of lovastatin upregulates BMP-2 and SOX9 expression and promotes chondrogenesis of rat caudal discs after needle puncture injury [290]. Simvastin promotes IVD repair processes, as evident in radiologic, histologic, and genetic assessments in a rat IVDD model [291]. Simvastin upregulates BMP2 expression and stimulates chondrogenic processes in experimental IVDD [292]. Rosuvastatin inhibits mechanical pressure-induced IVDD [293]. Rats fed a high-cholesterol diet display degenerative features in lumbar IVDs compared to rats fed a standard diet. This effect could be abolished by the cholesterol-lowering drug atorvastatin. Cholesterol levels are higher in NP cells treated with TNF-α and IL-1β, implicating cholesterol in the progression of IVDD, accelerated pyroptosis in NP cells, and ECM degradation. ER stress was responsible for this cholesterol-induced pyroptosis and ECM degradation [294].

## 9. Miscellaneous Plant Compounds as Prospective IVDD Treatment Agents

### 9.1. Aloin

**Figure 8:**
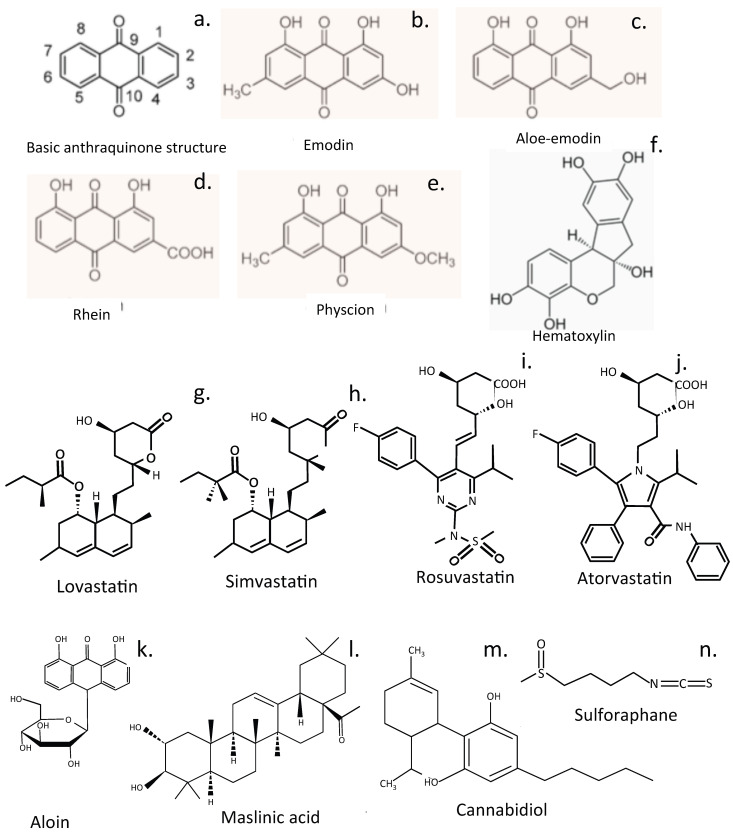
Miscellaneous compounds that have shown promise in the treatment of IVDD. Anthraquinone ring numbering (**a**), Anthraquinines (**b**–**e**), Emodin (**b**), Aloe-emodin (**c**), Rhein (**d**), Physcion (**e**), Hematoxylin (**f**), Statins (**g**–**j**), Lovostatin (**g**), Simvastin (**h**), Rosuvastatin (**i**), Atovastatin (**j**), Aloin (**k**), Maslinic acid (**l**), Cannabidiol (**m**) and Sulforaphane (**n**).

Aloin (barbaloin), an ancient traditional medicine, is a yellow-, orange-, and red-pigmented glycosylated anthraquinone from aloe [295]. It has been used to treat skeletal degenerative diseases [296], displays curative effects on ECM metabolism and apoptosis in TNF-*α*-treated NP cells, and inhibits oxidative stress and proinflammatory mediators suppressing the TGF-β-activated kinase 1 (TAK1)/NF-*κ*B pathway, downregulating the NLPR3 inflammasome in TNF-*α*-treated NP cells [93] (Figure 8).

### 9.2. Maslinic Acid

Maslinic acid (MA), an anti-inflammatory compound found in olive plants (*Olea europaea*) and many herbs mitigates cellular senescence, upregulates aggrecan and collagen II biosynthesis, and downregulates MMP and ADAMTS levels in NP cells. MA impedes the progression of IVDD in rat models through inhibition of the PI3K/AKT and NF-κB pathways. Molecular docking studies show that MA binds to PI3K, resulting in dysfunction of the PI3K/AKT pathway [73]. The health-promoting properties of olive oil that prevent a toxic build-up of saturated fatty acids in tissues reduces the risk of diseases linked to oxidative stress and chronic inflammation, where fatty acids are oxidized by ROS [297]. MA contributes to the tissue-protective properties of olive oil.

### 9.3. Cannabidiol

Cannabidiol reduces LBP by reducing inflammation, combats the anxiety associated with long-term LBP, and helps with sleep. In animal studies cannabidiol inhibited hydrogen peroxide-induced apoptosis [298], inflammation, and oxidative stress in NP cells, and showed tissue-protective properties in lesion-induced IVDD [299]. Cannabidiol reduced vertebral bone loss in rats with severe spinal cord injury.

### 9.4. Sulforaphane

Sulforaphane is an isothiocyanate polyphenolic compound that is generated in mustard and other cruciferous vegetables when tissue damage occurs. Glucoraphanin, a precursor of sulforaphane, is degraded by myrosinase, a β-thioglucosidase, to generate sulforaphane in plants [23]. Sulforaphane is also generated from glucoraphanin by intestinal bacteria and alleviates intestinal inflammation and oxidative stress, maintaining gut barrier integrity [300]. Sulforaphane delays IVDD by alleviating ER stress in NP cells [95] and also inhibits cellular senescence in the IVD [301].

## 10. The Role of Lipids in the Metabolism of Resident Disc Cell Populations

Excessive cholesterol levels have been shown to promote IVDD. Metabolic lipid disorders are associated with CEP senescence and calcification (EPC) in IVDD [302,303]. Oxidized low-density lipoprotein (ox-LDL) and lectin-like oxidized low-density lipoprotein receptor 1 (LOX-1) in human degenerative EPC is associated with hyperlipidemia (HLP) [304]. Hyperlipidemic conditions result in ox-LDL/LOX-1-induced EPC, mediated through LOX-1 receptor by the ROS/P38-MAPK/NF-κB cell signaling pathway. ox-LDL, formed by the lipid peroxidation of LDL, is a primary pathogenic factor in metabolic lipid disorders [305,306]. The LOX-1 receptor is the principal cell membrane receptor of ox-LDL. LOX-1 is expressed by IVD cells and is prominent in the CEP during EPC and IVDD [307]. Induction of EPC in rat IVDs by high-fat diets is mediated through the p38 MAPK/NF-κB cell signaling pathway [308]. HLP aggravates IVDD through the induction of inflammatory mediators and catabolic effects on NP and AF cells [309], promoting apoptosis in NP cells [59].

## 11. Pro-Resolving Anti-Inflammatory Lipids Rescue Degenerated IVDs

**Figure 9:**
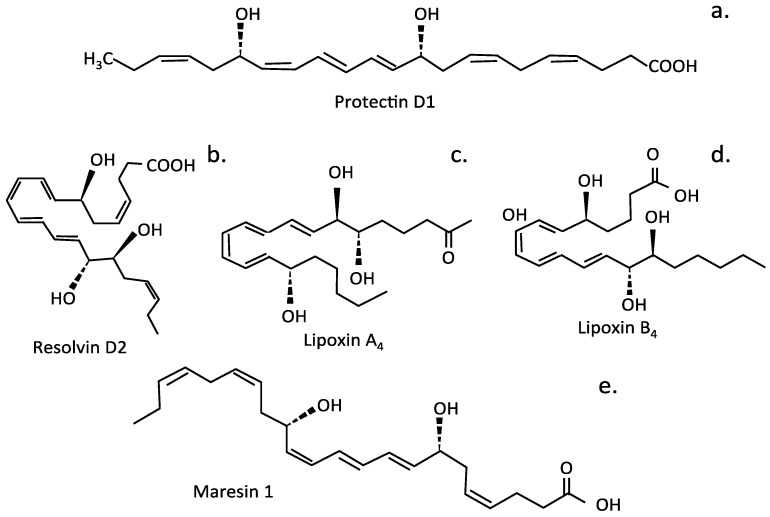
Bioactive lipid metabolites with properties conducive to the treatment of IVDD. Protectin D1 (**a**), Resolvin D2 (**b**), Lipoxin A_4_ (**c**), Lipoxin B_4_ (**d**), Maresin 1 (**e**).

While peroxidation of lipids can generate ROS-promoting inflammatory conditions in the IVD and degenerative tissue changes, some polyunsaturated IVD fatty acid lipid metabolites have been identified with potent anti-inflammatory properties (Figure 9). These include lipoxin A4 (LXA4), formed from AA (arachidonic acid); E series resolvins, formed from EPA (eicosapentaenoic acid); and D series resolvins, protectins, and maresins, formed from DHA (docosahexaenoic acid) [310]. These rescue the degenerated IVD by re-balancing lipid profiles. LXA_4_ inhibits ROS generation, NFkB activation, and the generation of pro-inflammatory IVD cytokines (e.g., IL8, IL13, IL12, and IL5) [311,312]. Intrathecal injection of LXA4 alleviated the development of neuropathic pain, inhibited the upregulation of pro-inflammatory cytokines (TNF-α and IL-1β), upregulated the expression of anti-inflammatory cytokines (TGF-β1 and IL-10), and attenuated the activation of NF-κB/p65, p-ERK, and p-JNK, but not that of p-p38, in a dose-dependent manner [313].

Resolvin D1 (RvD1) has potent anti-inflammatory and antinociceptive properties, alleviating neuropathic pain by regulating the inflammatory mediator NF-κB/p65 and p-ERK pathways [314]. Protectin PD1, an endogenous stereoselective lipid mediator, also has potent analgesic properties and regulated SIRT1-mediated CGRP signaling in a model of non-compressive lumbar disc herniation [315]. Maresin1 (MaR1), a macrophage-derived mediator of inflammation resolution, also displayed potent anti-inflammatory activity through inhibition of the NLRP3 inflammasome and NF-κB signaling [316]. LXA4, resolvins, protectins, and maresins inhibit the production and action of IL-6, TNF-α, and other pro-inflammatory cytokines. Therefore, they are of interest for the treatment of IVDD [313,314,317,318,319,320], as they can prevent excessive inflammation, restoring tissue homeostasis. Resolvin D2 (RvD2) suppresses the expression of pro-IL-1β, reducing the secretion of mature IL-1β by macrophages and deactivating the NLRP3 inflammasome. Injections of LXA4, resolvins, protectins, and maresins at sites of IVDD could be of significant therapeutic benefit.

A large number of compounds have been covered in this review; however, a few guiding comments are required to outline the most promising plant phytochemicals for the treatment of IVDD and potential IVD regeneration.

## 12. The Most Effective Plant Phytochemicals for Therapeutic Medical Applications

Flavonoids are a biodiverse family of bioactive plant phytochemicals that have, as a group, found widespread application in biomedicine, with over 10,000 flavonoids listed. It was beyond the scope of this study to cover all of these compounds. Selected flavonoid members that have shown promise in the treatment of IVDD have been covered in this review.

Quercetin, resveratrol, myrcetin, hyperoside, baicalein, kaempferol, luteolin, naringin, icariin, hesperidin, epigallocatechin 3-gallate (EGCG)/Urolithin A, and sesamin are all useful therapeutic plant phytochemicals that have been evaluated in the treatment of IVDD (Table 3). The properties of these compounds are briefly outlined in Table 3 and more extensively in Section 2.1.1, Section 2.1.2, Section 2.1.3 and Section 2.1.4 demonstrating why they have been evaluated in the treatment of IVDD and their potential to promote the regeneration of degenerated IVDs. These flavonoids have potent antioxidant and anti-inflammatory properties that counteract the changes induced in IVDs by NFkB cell signaling pathways, which play central roles in the generation of inflammatory and degradative pain-generating conditions in the IVD. Several flavonoid members also inhibit the expression of NFkB in disc tissues, and overlap between compounds classified as phenolics and flavonoids is evident. Curcumin and resveratrol are two phenolic compounds that display potent properties that counteract degradative events occurring in IVDD. Curcumin inhibits NFkB expression in IVD cells, produces anti-inflammatory and anti-catabolic conditions in IVDs, and counteracts chondrocyte apoptosis in the CEP. Sesamin also has CEP-protective properties, promoted by induction of PI3K/AKT/mTOR cell signaling. Myrcetin is a flavonoid and a phenolic compound that counteracts NFkB activity by promoting Nrf2/HO-1 cell signaling (Table 5). EGCG has potent regulatory properties over signaling pathways that control inflammation, oxidative stress, and apoptosis, providing protective effects over IVD cells. Resveratrol has potent antioxidant and anti-inflammatory properties in vitro and stimulates anabolic processes in IVDs, stimulating IVDD repair in vivo. Berberine is a promising alkaloid that ameliorates oxidation-induced apoptosis by modulating ER stress and autophagy in human NP cells. It also suppresses ECM degradation and IVDD by inhibiting the NFκB cell signaling pathway (Table 6).

Ginseng is a widely used herbal glycoside nutraceutical traditional Chinese medicine with antioxidant and anti-inflammatory properties. Ginsenoside Rg1 is of major interest for the treatment of IVDD since it regulates disc homeostasis, inhibits apoptosis, inflammation, and ECM degradation, delaying IVDD progression. Rg1 improves the proliferation of NP cells. Its IVDD inhibitory properties are affected through inhibition of the Wnt/β-catenin cell signaling pathway. Ginsenoside Rg1 also prevents activation of the NF-κB signaling pathway through the induction of IκK, inhibiting secretion of inflammatory mediators and stimulating aggrecan and collagen II biosynthesis. Ginsenoside Rg3 inactivates the p38 MAPK pathway, significantly reducing MMP2, MMP3, ADAMTS-4, and ADAMTS-5 degradative activity in the NP and restoring AF lamellar structure and function in the IVD composite structure (Table 7).

## 13. Key Cell Signaling Pathways Operative in IVDD

Mitochondrial dysfunction is critical in the pathogenesis of IVDD, influencing numerous cellular processes crucial for disc health. This dysfunction primarily results in excessive production of reactive oxygen species (ROS), leading to oxidative stress, mitochondrial DNA damage, and disrupted cellular bioenergetics [321]. These conditions activate apoptotic pathways, especially in NP cells, causing cell death and ECM degradation [322].

Enhanced mitophagy with agents such as urolithin A promotes mitochondrial homeostasis. The use of SIRT3 activators also protects cells from mitochondrial-induced damage [323].

Oxidative stress plays a central role in the development of IVDD by disrupting the balance between mitochondrial ROS production and the antioxidant defense system. In healthy IVDs, ROS levels are tightly regulated by antioxidants. However, in IVDD, aging, mechanical stress, and inflammation lead to an imbalance in ROS activity. This can cause cellular damage, lipid peroxidation, protein oxidation, and DNA damage, leading to apoptosis or dysfunction of NP and AF cells, critical for the maintenance of IVD structure and function.

Molecular pathways identified as key modulators of ROS in IVDD offer potential therapeutic targets. Manipulation of the activity of SIRT3 mitigates oxidative stress-induced senescence in NP cells [324]. The Keap1/Nrf2 axis plays a crucial role in the improvement of antioxidant defenses, providing protection against ROS-induced damage [324,325]. Activation of this pathway counteracts oxidative stress and slows IVDD processes. Hesperidin and glycitin modulate specific cellular signaling pathways, mitigating oxidative damage within the IVD [322,326]. Therapeutic strategies that reduce oxidative stress and enhance cellular antioxidant activity are promising strategies to inhibit IVDD and promote normal IVD functions.

### 13.1. Nrf2 Cell Signaling and IVDD

**Figure 10:**
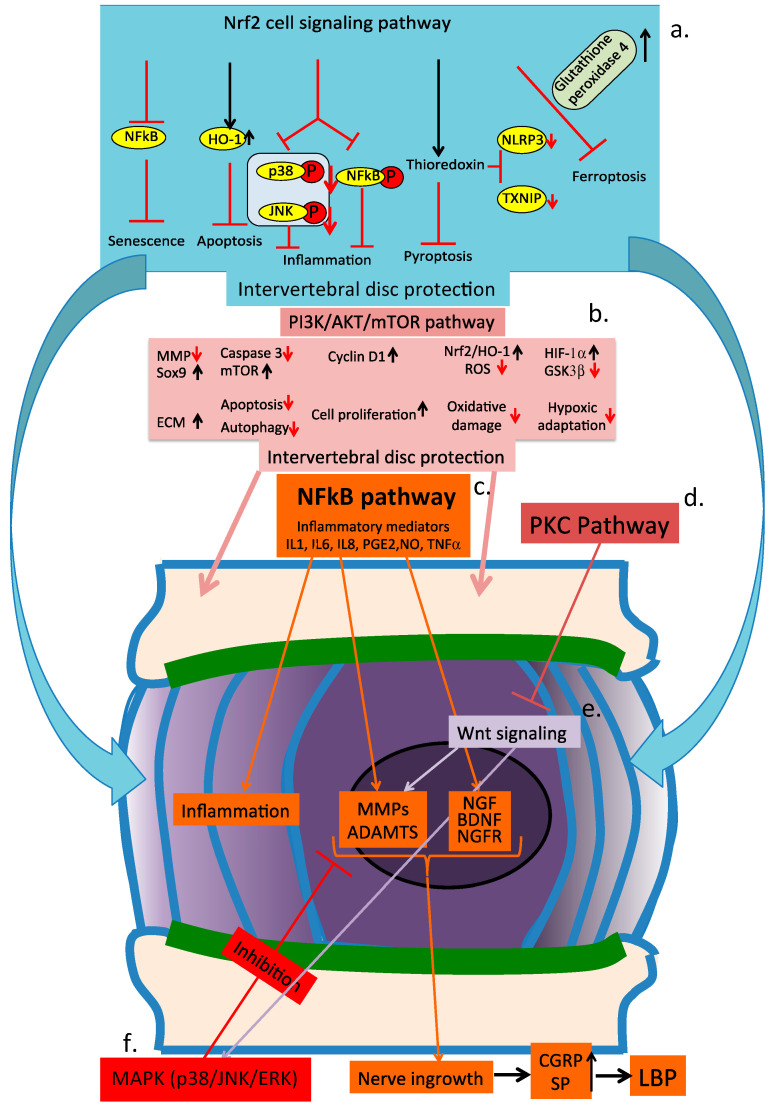
Schematic of the major cell signaling pathways of the intervertebral disc operating during disc degeneration. (**a**) Nrf2 cell signaling pathway, (**b**) PI3/AKT/mTOR pathway, (**c**) NFκB pathway, (**d**) PKS pathway, (**e**) Wnt cell signaling pathway, and (**f**) MAPK (p38/JNK/ERK) cell signaling pathway.

As shown in Figure 10, nuclear factor erythroid 2-related factor 2 (Nrf2), is an important antioxidant transcription factor that plays a crucial role in the modulation of the pathogenesis and progression of IVDD. It maintains redox homeostasis by protecting NP cells from oxidative stress, damage through inflammatory processes, ECM degradation, cell senescence, cell death, and the generation of LBP [175]. Nrf2 is a master antioxidant transcription factor with protective properties that counteract oxidative stress by mediating cellular damage in IVDD. Nrf2 is negatively regulated by Kelch-like ECH-associated protein 1 (Keap1). Nrf2 regulates the transcription of downstream antioxidant genes expressed by disc cells by binding to antioxidant response elements (AREs) in promoter regions in genes, such as heme oxygenase-1 (HO-1), glutathione (GSH), superoxide dismutase (SOD), catalase (CAT), and NADPH quinone dehydrogenase 1 (NQO1) [327]. This antioxidant defense system regulates cell apoptosis, senescence, ECM turnover, inflammatory responses displayed by NP cells, autophagy, and calcification of the CEP in IVDD [328]. Heme oxygenase-1 (HO-1) has antioxidant, anti-inflammatory, and anti-apoptotic protective properties regulated by Nrf2 and plays a particularly important role in the protection of IVD cells during IVDD [329]. Sulforaphane (SFN) is a natural compound found in the Brassica plant family that displays potent Nrf-2 agonist activity. It also has antioxidant properties in vitro and in vivo that counteract the degradative events that occur in IVDD [95]. SFN can promote the entry of Nrf-2 into the nucleus and increase the expression level of heme oxygenase 1 (HO-1) in vitro. This aids in the clearance of ROS accumulation in IVD cells, which can induce ER stress, thereby delaying the progression of IVDD.

A widespread range of phenolic phytochemicals display potent antioxidant and anti-inflammatory properties that protect IVD tissues from damage during IVDD [330]. Many of these plant phenolic phytochemicals also promote expression of the Nrf2/Kelch system, providing cell and tissue protection in IVDD [331,332,333]. The Nrf2 cell signaling pathway inhibits cellular senescence, apoptosis, and inflammation.

Some examples of these phenolic compounds have been discussed earlier in this review, including quercetin, curcumin, icariin, myrcetin, epigallocatechin 3-gallate (EGCG), baicalein, resveratrol, and SFN; however, this is an extremely diverse group of compounds, with 10,000 flavonoids listed alone [330,331].

### 13.2. PI3/AKt/mTOR Cell Signaling in IVDD

Activation of the phosphatidylinositol 3-kinase (PI3K)/Akt/mTOR pathway also has cell-protective properties through inhibition of IVDD, producing an increase in ECM synthesis, inhibition of cell apoptosis, promotion of cell proliferation, induction or prevention of cell autophagy, reduced oxidative damage, and facilitative adaptation to a hypoxic microenvironment [334]. A large number of plant phytochemicals can activate the PI3K/Akt cell signaling pathway, including quercetin, resveratrol, curcumin, proanthocyanidins, kaempferol, tyrosol, icariin, naringin, myrcetin, curcumin, hesperidin, and baicalein, indicating that these have potential in the treatment of IVDD [24,334].

### 13.3. NFkB Cell Signaling Plays a Central Role in Inflammatory Processes in IVDD

The nuclear factor NF-kappa B cell signaling pathway is central to inflammation in the IVD. NF-kappa B regulates the expression of proinflammatory genes controlling the synthesis and secretion of cytokines, chemokines, and adhesion molecules [335]. Persistent inflammation is associated with IVDD due to prolonged nuclear factor κB (NFκB) activation and leads to recruitment of active macrophages [336]. Macrophages have important roles in inflammation-driven IVDD, stemming from conversion to a catabolic phenotype [337]. M2 macrophages promote inflammatory processes in the IVD and regulate IVD cellular activities, affecting IVD ECM synthesis and degradation, vascularization, and innervation in the IVD, which contributes to the progression of IVDD and generation of LBP. The NFκB family of transcription factors regulate immune development, immune responses, inflammation, and cancer through interactions between NFκB dimers, IκB regulators, and IKK complexes [338]. The IKK complex is a central regulator of NFkB activation [339]. The IKK complex consists of two kinases (IKKalpha and IKKbeta) and a regulatory subunit, NEMO/IKKgamma [340]. IKKalphaand IKKbeta mediate phosphorylation of IkB proteins in signal transduction pathways that lead to NFkB activation. IKKbeta(and IKKgamma) have essential roles in the rapid activation of NFkB in response to proinflammatory signaling cascades, triggered by TNF alpha or LPS. Related IKKs bearing structural similarity to IKKalpha and IKKbeta are important for the activation of interferon response factor 3 (IRF3) and IRF7, transcription factors that play key roles in the induction of type I interferon (IFN-I). NF-kB is a cytoplasmic transcription factor, which, upon activation, translocates to the nucleus, where it controls the expression of 400 different genes, making it a master regulator of inflammatory processes and innate and adaptive immune responses. A wide range of plant phenolic phytochemicals can inhibit the activation of NFkB, controlling inflammatory processes and the degradative changes they induce in IVDD [330,341].

### 13.4. MAPK (p38, ERK, JNK) Cell Signaling in IVDD

This review has shown that in IVDD, elevated expression and synthesis of MMPs and inflammatory mediators is mediated by NFkB- and MAPK-regulated pathways [342,343]. NFkB and MAPK are considered major regulators of inflammation and catabolism in processes that lead to IVDD [344]. c-Jun N-terminal kinase (JNK) is a key branch of the MAPK signaling pathway [345] in IVDD [346] and interacts with the PI3K/Akt and NF-ĸB cell signaling pathways, influencing cell growth, survival, and metabolism [347,348,349].

Baicalin [350], berberine [246], glycyrrhizin [351], sesamin [188], and crocin [118] all inhibit aspects of JNK cell signaling. Flavonoids (e.g., quercetin, hyperoside), glycosides (e.g., ginsenosides, notoginsenosides), terpenoids (e.g., aucubin, celastrol), phenolic compounds (e.g., curcumin, resveratrol), and alkaloids (e.g., berberine) all exert therapeutic effects on IVDD by modulating key signaling pathways, including Sirtuin-1 (SIRT1), MAPK, PI3K/Akt, and Nrf2 [175], thus providing potent anti-inflammatory, antioxidant, anti-apoptotic, anti-senescence, and IVD regenerative properties [24,341]. NFκB signaling is associated with pain-related neuropeptide expression and pain generation in IVDD [352,353]. Inflammatory mediators induced by NFkB, such as IL-1beta and TNF alpha stimulate production of NGF by IVD cells [45,354]. NGF, stimulate IVD cells, resulting in ECM degradation and the promotion of IVDD. NGF levels are elevated in degenerated IVDs compared to normal IVDs [355]. BDNF levels are also elevated in degenerated IVDs [44].

### 13.5. PKC Signaling Inhibits Wnt-Mediated Processes in IVDD

Activation of PKC signaling leads to an increase in ECM synthesis and cell proliferation, inhibiting IVD degeneration through inhibition of Wnt signaling [356]. These processes are summarized in Figure 10.

## 14. Limitations on the Therapeutic Application of Plant Phytochemicals

### Bioavailability of Plant Compounds

Phytochemicals hold immense potential for improving health; however, they face challenges that limit their application in mainstream therapeutics and use as dietary supplements due, in some cases, to their low bioavailability, poor solubility, and stability. The full potential of polyphenolic and carotenoid phytochemicals is therefore challenged, preventing their full therapeutic potential from being realized [357]. Future research should further investigate the role of gut microbiota and how they influence the bioavailability of dietary plant phytochemicals. The gut microbiome can process phytochemicals into fragments such as urolithin A, retaining the bioactivity of the native molecule that is more easily transported and making them more bioavailable [46]. Polyphenols, isothiocyanates, and curcumin can modulate the gut microbiota, impacting health outcomes. Polyphenols promote beneficial bacteria, such as *Bifidobacterium* and *Lactobacillus*, improving gut and metabolic health-reducing inflammation in obesity and diabetes [358]. Furthermore, isothiocyanates found in cruciferous vegetables support gut microbe diversity and reduce cancer risk by modulating gut inflammation [359]. Moreover, curcumin impacts the gut–brain axis, reducing neuroinflammation in neurodegenerative diseases like Alzheimer’s [360] and Parkinson’s [361,362,363]. Understanding the interaction between phytochemicals and gut microbiota could unveil new therapeutic avenues, leading to personalized nutritional strategies based on individual metabolic profiling. The inclusion of phytochemical supplements, functional foods, and dietary interventions may make it possible to realize the health benefits of phytochemicals in a secure and efficient manner.

## 15. Effective Delivery of Phytochemicals into the IVD

This review has established the chemical properties of plant compounds with antioxidant and anti-inflammatory properties with potential application in IVDD. However, a critical step that needs to be resolved is how these compounds are delivered to the IVD. Degenerated IVDs have depleted levels of aggrecan, their main space-filling proteoglycan, making them more amenable to the diffusive entry of metabolites. Blood vessels penetrate deeper into degenerated IVDs since the internal pressure is lower than in normal IVDs, where blood vessels do not penetrate past the outermost lamellar layer in the annulus. The elevated ingrowth of blood vessels into degenerated IVDs represents a potential entry route for plant compounds carried in the blood stream to central regions of the IVD, where tissue damage may be advanced. Further studies need to be undertaken to determine the diffusive properties of IVDs to plant compounds to develop an effective means of administering these into the IVD.

A gut–IVD axis has been established, with blood networks supplying the lumbar spinal region serving as conduits for phytochemicals to reach degenerated IVDs [364]. Innovative delivery systems, such as nanoparticles, liposomes, and micro-encapsulation, also show promise in enhancing the absorption and stability of administered phytochemicals, aiding their full therapeutic potential. Multifunctional stimuli-responsive nanoparticles show potential for their capacity to provide precise targeting and controlled therapeutic release of cargo chemicals, offering improved localization and sustained delivery of phytochemicals into the IVD [365]. This may overcome the limitations of conventional therapeutic treatment modalities, enabling more effective, targeted management strategies for IVDD [366]. Immune-defense microspheres have emerged with critical properties that inhibit IVDD through modulation of the inflammatory microenvironment, while also promoting disc regeneration [367]. Nanocomposite EGCG-coated hydroxyapatite composites with O-carboxymethyl chitosan cross-linked to HA have proved useful for this application.

## 16. Future Research on Plant Phytochemicals

**Figure 11:**
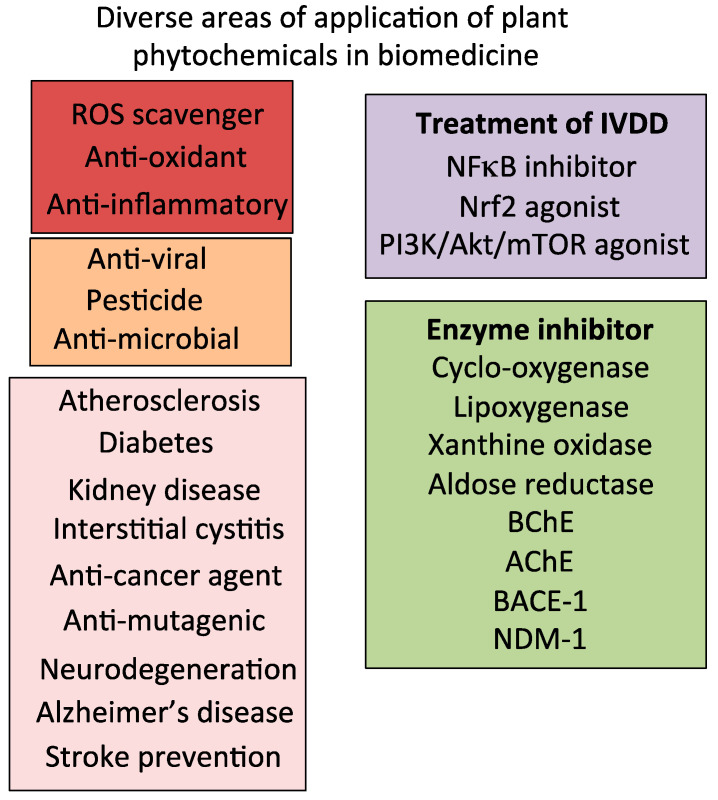
Some examples of the diverse areas in biomedicine where plant phytochemicals or drugs developed from these have found therapeutic application. **Abbreviations**: ROS, reactive oxygen species; BChE, butyrylcholinesterase, serine hydrolase; AChE, acetylcholinesterase; BACE-1, beta-secretase 1, also known as beta-site amyloid precursor protein cleaving enzyme 1; NDM-1, New Delhi metallo beta lactamase.

Nature has provided a highly diverse array of molecules in plants, which are an immense resource. Plant compounds have diverse properties and have been harnessed in medical applications to combat disease for centuries. It should be noted that the origins of many successful present-day pharmaceuticals had their origins in plant compounds that were noted to have bioactive properties of potential application in biomedicine [28,29]. A total of 80% of 122 plant-derived drugs are reported to have had their origins in ethnopharmacological folkloric medicine [368]. The biodiversity of these compounds and their many applications in biomedicine are clearly evident. Flavonoids in particular have many potential applications in biomedicine, but this is only one class of compounds covered in this review (Figure 11). This is an area that warrants further investigation in the future. History shows the likelihood of this being a fruitful area of investigation, particularly in problematic present-day medicinal areas, such as antiviral development. This may potentially uncover compounds that may be used in a preventative capacity rather than more costly approaches requiring the development of vaccines and therapeutic antibodies to treat viral symptoms. Pentosan polysulfate, a sulfated semi-synthetic plant polysaccharide, is an example of a cost-effective pan-specific antiviral compound that prevents SARS CoV-2 infections [369]. It also has pleiotropic cell and tissue-protective properties of therapeutic application in long COVID disease [370].

Other areas that warrant further research are optimized phytochemical delivery systems to the IVD, the impact of the gut microbiome on the processing of dietary plant phytochemicals, and how this effects the delivery of bioactive phytochemical species into the IVD via the gut–IVD axis. For example, a study using a smart responsive injectable hydrogel loaded with icariin has just been published, and similar delivery systems are expected to follow in the near future [371]. This delivery system with icariin inhibited ferroptosis and promoted regeneration of the NP.

## Figures and Tables

**Table 1:** IVDD signaling pathways that interventional plant compounds affect.

PI3K/AKT/mTOR	[52,53,54,55,56,57]
ERK	[58]
MAPK	[43,59,60,61,62,63,64]
JAK/STAT	[65,66,67]
AMPK	[68,69,70]
PI3K/Akt	[40,52,57,71,72,73,74,75,76,77]
Nrf2/NFkB	[39,78,79,80,81,82,83]
Wnt	[84,85]
SIRT3/mitophagy	[86,87,88,89]
ER stress	[69]
TLR/M2	[90]
TAK1-mediated PANoptosis	[91,92,93]
TLR4	[63,94]
Nrf2/HO-1	[42,95,96]

**Table 2:** Some examples of the therapeutic properties of plant compounds.

Compound	Main Cell Signaling Pathways Affected	Therapeutic Effects
Flavonoids	NF-kB	Inflammation ↓
Terpenoids	NF-kB PI3K/Akt	Inflammation ↓ ECM stabilization ↑
Glycosides	SIRT/Nrf2	Apoptosis ↓
Phenolics	SIRT/Nrf2	Apoptosis ↓
Alkaloids	MAPK (p38/JNK/ERK) AMPK/mTOR	Cell viability ↑ Autophagy ↑

Abbreviations: NF-kB, nuclear factor kappa-light-chain-enhancer of activated B cells; SIRT, sirtuin 1, NAD-dependent deacetylase; Nrf2, nuclear factor erythroid 2-related factor 2; PI3K, phosphatidylinositol 3-kinase; Akt, protein kinase B; MAPK, mitogen-activated protein kinase; p38, mitogen-activated protein kinase; JNK, c-Jun N-terminal kinase; ERK, extracellular signal-regulated kinase; AMPK, adenosine monophosphate-activated protein kinase; mTOR, mammalian target of rapamycin.

## Data Availability

No new data was generated in this study, all data is directly available from the cited references.

## Abbreviations

IVDD	Intervertebral disc degeneration
ADAMTS4	A disintegrin and metalloproteinase with thrombospondin motifs 4
Akt	Protein kinase B
AMPK	5′ AMP-activated protein kinase; iNOS, inducible nitric oxide synthase.
AMP	Adenosine monophosphate
ARE	Antioxidant response elements
BDNF	Brain-derived neurotrophic factor (abrineurin)
BECN2	Macroautophagy and autophagy family member beclin 2 (Bcl2/Bax)
BMP-2	Bone morphogenetic protein-2
CDK	Cyclin-dependent kinase
cGAS/Sting	Cyclic GMP-AMP synthase–stimulator of interferon genes
COX-2	Cyclooxygenase-2, prostaglandin–endoperoxide synthase 2
E2	Prostaglandin E2
EGCG	Epigallocatechin gallate
ER	Endoplasmic reticulum
ERK	Extracellular signal-regulated kinase
FOXO3	Forkhead box O3 protein
GPRASP1	G protein-coupled receptor associated sorting protein 1
PI3K	Phosphatidylinositol 3-kinase
HDL	High-density lipoprotein
HO-1	Heme oxygenase-1
IL-6	Interleukin 6
IL-1β	Interleukin-1 beta
iNOS	Inducible nitric oxide synthase
ITGA2	Integrin alpha 2
JAK	Janus kinase
JNK	c-Jun N-terminal kinase
Keap-1	Kelch-like ECH-associated protein 1
LDL	Low-density lipoprotein
LPS	Lipopolysaccharide
MAPK	Mitogen-activated protein kinase
MMP3	Matrix metalloprotease 3
mTOR	Mammalian target of rapamycin.
MS	Muscular sclerosis
NADQO	NAD(P)H:quinone oxidoreductase 1
NF-kB	Nuclear factor kappa-light-chain-enhancer of activated B cells
P38	Mitogen-activated protein kinase
P53	Tumor protein p53, a regulatory transcription factor protein
Nrf2	Nuclear factor erythroid 2-related factor 2
PPAR	Protease activated receptor
PANoptosis	A prominent innate immune, inflammatory, and lytic cell death pathway initiated by innate immune sensors driven by caspases and receptor-interactive protein kinases
RA	Rheumatoid arthritis
RIP3	Receptor-interacting serine/threonine-protein kinase 3
ROS	Reactive oxygen species
SIRT-1	Sirtuin 1, NAD-dependent deacetylase
SLE	Systemic lupus erthythematosus
SOX6	SRY box 6 transcription factor
SDF-1	Stromal-derived factor
STAT	Signal transducer and activator of transcription
TAK1	Mitogen-activated protein kinase kinase kinase 7(Map3k7)
TLR	Toll-like receptor
TLR4	Toll-like receptor 4
TLR4/MD-2	Toll like receptor-4/Muscarinic acetylcholine receptor-2 complex
TNF-α	Tumor necrosis factor alpha
VLDL	Very-low-density lipoprotein
Wnt	Wingless-type MMTV integration site family
